# Neuropeptide and Small Transmitter Coexistence: Fundamental Studies and Relevance to Mental Illness

**DOI:** 10.3389/fncir.2018.00106

**Published:** 2018-12-21

**Authors:** Tomas Hökfelt, Swapnali Barde, Zhi-Qing David Xu, Eugenia Kuteeva, Joelle Rüegg, Erwan Le Maitre, Mårten Risling, Jan Kehr, Robert Ihnatko, Elvar Theodorsson, Miklos Palkovits, William Deakin, Gyorgy Bagdy, Gabriella Juhasz, H. Josée Prud’homme, Naguib Mechawar, Rochellys Diaz-Heijtz, Sven Ove Ögren

**Affiliations:** ^1^Department of Neuroscience, Karolinska Institutet, Stockholm, Sweden; ^2^Department of Neurobiology, Beijing Key Laboratory of Neural Regeneration and Repair, Beijing Laboratory of Brain Disorders (Ministry of Science and Technology), Beijing Institute for Brain Disorders, Capital Medical University, Beijing, China; ^3^Department of Clinical Neuroscience, Karolinska Institutet, Stockholm, Sweden; ^4^The Center for Molecular Medicine, Stockholm, Sweden; ^5^Swedish Toxicology Sciences Research Center, Swetox, Södertälje, Sweden; ^6^Pronexus Analytical AB, Solna, Sweden; ^7^Department of Physiology and Pharmacology, Karolinska Institutet, Stockholm, Sweden; ^8^Department of Clinical Chemistry, Linköping University, Linköping, Sweden; ^9^Department of Clinical and Experimental Medicine, Linköping University, Linköping, Sweden; ^10^Department of Anatomy, Histology and Embryology, Semmelweis University, Budapest, Hungary; ^11^Neuroscience and Psychiatry Unit, University of Manchester, Manchester, United Kingdom; ^12^Department of Pharmacodynamics, Semmelweis University, Budapest, Hungary; ^13^MTA-SE Neuropsychopharmacology and Neurochemistry Research Group, Hungarian Academy of Sciences, Semmelweis University, Budapest, Hungary; ^14^NAP 2-SE New Antidepressant Target Research Group, Hungarian Brain Research Program, Semmelweis University, Budapest, Hungary; ^15^SE-NAP2 Genetic Brain Imaging Migraine Research Group, Hungarian Brain Research Program, Semmelweis University, Budapest, Hungary; ^16^Douglas Hospital Research Centre, Verdun, QC, Canada; ^17^Department of Psychiatry, McGill University, Montreal, QC, Canada

**Keywords:** allostatic load, epigenetics, galanin, locus coeruleus, major depression disorder, neuropeptides, resilience

## Abstract

Neuropeptides are auxiliary messenger molecules that always co-exist in nerve cells with one or more small molecule (classic) neurotransmitters. Neuropeptides act both as transmitters and trophic factors, and play a role particularly when the nervous system is challenged, as by injury, pain or stress. Here neuropeptides and coexistence in mammals are reviewed, but with special focus on the 29/30 amino acid galanin and its three receptors GalR1, -R2 and -R3. In particular, galanin’s role as a co-transmitter in both rodent and human noradrenergic locus coeruleus (LC) neurons is addressed. Extensive experimental animal data strongly suggest a role for the galanin system in depression–like behavior. The translational potential of these results was tested by studying the galanin system in *postmortem* human brains, first in normal brains, and then in a comparison of five regions of brains obtained from depressed people who committed suicide, and from matched controls. The distribution of galanin and the four galanin system transcripts in the normal human brain was determined, and selective and parallel changes in levels of transcripts and DNA methylation for galanin and its three receptors were assessed in depressed patients who committed suicide: *upregulation* of transcripts, e.g., for galanin and GalR3 in LC, paralleled by a *decrease* in DNA methylation, suggesting involvement of epigenetic mechanisms. It is hypothesized that, when exposed to severe stress, the noradrenergic LC neurons fire in bursts and release galanin from their soma/dendrites. Galanin then acts on somato-dendritic, inhibitory galanin autoreceptors, opening potassium channels and inhibiting firing. The purpose of these autoreceptors is to act as a ‘brake’ to prevent overexcitation, a brake that is also part of *resilience* to stress that protects against depression. Depression then arises when the inhibition is too strong and long lasting – a maladaption, allostatic load, leading to depletion of NA levels in the forebrain. It is suggested that disinhibition by a galanin antagonist may have antidepressant activity by restoring forebrain NA levels. A role of galanin in depression is also supported by a recent candidate gene study, showing that variants in genes for galanin and its three receptors confer increased risk of depression and anxiety in people who experienced childhood adversity or recent negative life events. In summary, galanin, a neuropeptide coexisting in LC neurons, may participate in the mechanism underlying resilience against a serious and common disorder, MDD. Existing and further results may lead to an increased understanding of how this illness develops, which in turn could provide a basis for its treatment.

## Introduction

The first evidence for chemical signaling in the central nervous system was reported by Eccles et al. (1954), when they demonstrated that acetylcholine is the transmitter released from motor neuron collaterals onto Renshaw cells in the spinal cord. Some 10 years later the Canadian electrophysiologist Hugh McLennan in his monograph “Synaptic transmission” (McLennan, 1963) reviewed in some detail the evidence for a number of molecules being transmitters: “Acetylcholine,” “Catecholamines,” “5-Hydroxytryptamine,” “Substance P,” “Factor I and the Inhibitory Transmitter,” “GABA and Glutamic Acid,” and “Cerebellar Excitatory Factor” were the chapter sub-headings. Some further compounds were mentioned, like other amino acids. A detailed table of the regional distribution of these molecules was included. In the “Conclusions” McLennan stated “With the exception of a number of cholinergic and rather fewer adrenergic systems, the data supporting a certain type of chemical mediation in any given situation are quite inadequate, and in spite of the inherent difficulties the number of problems to be solved are of great interest.” Indeed, many efforts in the following years rapidly expanded the number of candidates and ‘certified’ their transmitter status – work still ongoing. However, to identify a molecule as a transmitter was at that time often a difficult process with strong pro and contra arguments. More recently completely different molecules have appeared on the scene, not stored in vesicles and thus not exocytosed, like nitric oxide (NO) and hydrogen sulfide (H_2_S), sometimes called “gasotransmitters” (Paul and Snyder, 2015). Subsequently, substance P, mentioned already by McLennan, was identified as a member of the by far most diverse group of signaling molecules (>100) in the nervous system, the neuropeptides (Burbach, 2010).

The purpose of the present article is to review data on one of these peptides, galanin, which was discovered by Tatemoto et al. (1983) at Karolinska Institutet, a peptide that is a co-transmitter in many systems. In particular, focus is on recent results describing the distribution of galanin and it three receptors GalR1-3 in the ‘normal’ human brain by studying post mortem tissue samples (Le Maitre et al., 2013). More importantly, results are discussed showing significant changes in expression of the galanin family ‘members’ in post mortem brains from depressed patients having committed suicide, as compared to controls (Barde et al., 2016). A hypothesis is presented on a possible role of galanin, coexisting in noradrenergic neurons in the locus coeruleus (LC), in the development of depression and in resilience. This hypothesis is based on results from extensive animal experiments, so discussion of the human studies is preceded by an overview of “neuropeptides” with some comments on “methodological approaches,” of “neuropeptide – small transmitter molecule coexistence,” of the neuropeptide “galanin,” followed by a summary of the critical and relevant animal experiments.

## Neuropeptides

The concept of neuropeptide transmitters was introduced by the late Dutch scientist David de Wied and colls. (see De Wied and De Kloet, 1987). Neuropeptides are different from classic transmitters in several ways (Strand, 1991). In brief, neuropeptides are ribosomally synthesized as large precursor molecules in cell soma and dendrites (Noda et al., 1982; Mains et al., 1987), and the bioactive peptide(s) is excised from prepropeptide precursors by convertase enzymes (Seidah and Chretien, 1999). Packed in storage vesicles the peptides are axonally transported and released by exocytosis from nerve terminals, and also from dendrites and soma.

Neuropeptides in the nervous system encompass > 100 members (Burbach, 2010), almost always acting via one or more of a correspondingly large number of 7-transmembrane, G protein-coupled receptors (GPCRs) (>200). Much research is ongoing in the neuropeptide field. A search on PubMed with the terms “neuropeptides, review” (August 1, 2018) generated 35.579 hits. However, work on neuropeptides has not been without controversies. Already in the 1990’ies doubts were expressed with regard to functional significance [see for example the article entitled “Superfluous neurotransmitters” (i.e., *neuropeptides*) by Bowers (1994)]. The recent statement by Sudhof (2017) still reflects a cautious attitude: “At the forefront of early molecular neuroscience was the identification of neuropeptide precursors and neuropeptide receptors (Noda et al., 1982), but since then the question of neuropeptide signaling has largely faded from view with a few exceptions.”

However, peptides have an important and well accepted physiological function, when they are expressed in neurosecretory systems (Scharrer and Scharrer, 1937; Bargmann, 1949; Bargmann and Scharrer, 1951; Swaab et al., 1975; Vandesande and Dierickx, 1975; Brownstein and Mezey, 1986; Swanson et al., 1986; Ceccatelli et al., 1989; Meister, 1993; Morris et al., 1998; Gainer et al., 2002; Landgraf and Neumann, 2004; Jurek and Neumann, 2018), releasing their peptides into the general circulation (e.g., vasopressin, oxytocin) (Acher and Chauvet, 1954; Du Vigneaud, 1954), or into the hypothalamic portal circulation [thyrotropin releasing hormone (TRH), luteinizing releasing hormone (LHRH), somatostatin (a.k.a. growth hormone release-inhibiting hormone, GHR-IH), corticotropin releasing factor/hormone (CRF/CRH), and growth hormone releasing hormone (GHRH)] (Guillemin, 1978; Schally et al., 1978; Spiess et al., 1981, 1983; Vale et al., 1981; Brazeau et al., 1982; Rivier et al., 1982).

It is fair to say that many of the initial, high expectations of neuropeptides were not met. Examples are: (i) the discovery of the first endogenous ligands met- and leu-enkephalin for the morphine receptor (Hughes et al., 1975), present in dorsal horn interneurons (Hokfelt et al., 1977b), was expected to lead to new efficacious medicines for fighting pain, without the serious side effects of morphine; and (ii) antagonists to substance P, present in sensory neurons and the spinal dorsal horn (Lembeck, 1957; Hokfelt et al., 1975b; Takahashi and Otsuka, 1975) and acting as a transmitter (Otsuka et al., 1975; Henry, 1976) via NK1 receptors (Mantyh et al., 1995), were anticipated to represent a new type of painkiller.

These ‘failures’ have occurred in spite of considerable efforts from academia and pharmaceutical companies. For example, a substance P (neurokinin 1, NK1) antagonist was tested some 25 years later in the clinic but did not induce analgesia (Hill, 2000; Herbert and Holzer, 2002). However, and interestingly, it was also reported in a placebo-controlled trial in patients with moderate to severe major depression that the substance P (NK1) antagonist MK-869 (Aprepitant, EMEND), has robust antidepressant activity (Kramer et al., 1998). Moreover, the improvement was similar to that observed (in the same study) with the widely used antidepressant serotonin reuptake inhibitor (SSRI) paroxetine (Paxil, Seroxat) and essentially without (the common sexual) side effects seen with SSRIs (Kramer et al., 1998). However, a phase 3 trial failed to reproduce the antidepressant effects of MK-869 (Keller et al., 2006). Reasons for the failure in the treatment of depression have recently been analyzed (Rupniak and Kramer, 2017), and psychiatric studies of NK1 are still ongoing (e.g., Frick et al., 2016; Schank and Heilig, 2017). Neuropeptides and pharmacotherapy for depression will be discussed further below.

There is, however, one ‘sphere’ where neuropeptides have achieved a significant ‘status,’ and that is as markers for specific neuron populations, in particular in cortex and hippocampus^1^, without defining their functional role. This said, there *are* interesting examples, where a neuropeptide is essential for particular mouse behaviors. For example, in the lateral amygdaloid nucleus gastrin releasing peptide (GRP) regulates fear via the GRP receptor (Shumyatsky et al., 2002), and the same peptide and receptor modulate sighing in the preBötzinger complex in the ventrolateral medulla oblongata (Li et al., 2016). Arcuate AgRP neurons projecting to i.a. the parabrachial nucleus (Broberger et al., 1998) represent another example. These neurons are GABAergic and also express and release NPY, thus a good example of peptide and small molecule co-transmission. Alhadeff et al. (2018) have now shown that, of these three molecules, NPY via its NPY Y1 receptor is selectively responsible for a pain-inhibiting effect. Finally, based on a Drosophila study (Asahina et al., 2014), Zelikowsky et al. (2018) use a battery of the most recent methodologies to conduct a landmark study that demonstrates a key role for the neuropeptide tachykinin 2/neurokinin B and its receptor NK3 in chronic isolation stress, opening up for a new treatment strategy of this serious mood disorder.

The therapeutic potential of neuropeptide signaling has been extensively discussed based on animal experiments. These experiments also consider a possible role of neuropeptides in behaviors related to stress and mood regulation, and explore their receptors as possible targets for antidepressant drug development, a main theme of this review (Herbert, 1993; Maubach et al., 1999; Hokfelt et al., 2003; Holmes et al., 2003; Sajdyk et al., 2004; Nemeroff and Vale, 2005; Millan, 2006; Steckler, 2008; Wu et al., 2011; Griebel and Holsboer, 2012; Griebel and Holmes, 2013).

## Localization and Function of Neuropeptides: Methods

Four methods are of crucial importance for the exploration of neuropeptides and their coexistence with small molecule transmitters: Immunohistochemistry (IHC), radioimmunoassay (RIA), *in situ* hybridization (ISH) and real-time (quantitative) polymerase chain reaction (qPCR).^2^ These methods allow not only studies of the localization and levels of various neuropeptides but also give a hint toward functionality.

Neuropeptides released from nerve endings have to be replaced by ribosomal synthesis in cell soma followed by axonal transport. Thus, replacement can take a considerable time, of course especially in neurons with long projections, and especially in large brains like the human brain. However, here dendritic release is special as the distance between site of release and site of synthesis is short and allows for rapid replacement. In fact, dendritic release is associated with distinct features: peptide release (see below) via exocytosis is stimulated by depolarization-induced Ca2+ entry through voltage-gated calcium channels, whereby the SNARE proteins in the dendrites may partly differ from those in nerve endings (Ludwig and Leng, 2006; Kennedy and Ehlers, 2011; Ovsepian and Dolly, 2011; van den Pol, 2012; Ludwig et al., 2016).

Neuropeptide dynamics distinctly contrast those of classic transmitters: the latter are enzymatically produced also at release sites (in the nerve endings), and they have a membrane reuptake mechanism (transporters) at both the cell and storage vesicle membrane (Kanner, 1994; Liu and Edwards, 1997; Chen et al., 2004; Eiden et al., 2004; Hahn and Blakely, 2007; Torres and Amara, 2007). These transporters allow rapid replacement at the site of release, i.e., no axonal transport is needed. Such transporters have not been demonstrated for neuropeptides. This said, there is evidence that galanin after intraventricular injection can accumulate in a small number of neurons, e.g., in the hippocampus (Jansson et al., 2000).

Monitoring peptide mRNA levels with ISH provides a measure of activity of specific neurons. If analyzed in an experimental paradigm, one may even associate involvement of a peptide with a certain function. For example, an increase in galanin transcripts in dorsal root ganglion (DRG) neurons, after peripheral nerve injury, has been interpreted as a defense against pain (Xu et al., 2008) and as a signal for repair (Hobson et al., 2010).

However, reporting of mRNA levels alone always raises the issue of translation: Can the presence of transcript really equal the presence of protein (peptide)? Many studies suggest this to be the case in DRGs, for example. Also, the experiments on human *postmortem* brains, where transcript (qPCR) and peptide (RIA) were analyzed in the same samples (Barde et al., 2016) support this view (see below). Ideally this issue can be solved by double-labeling of individual cells: ISH for transcript and IHC for neuropeptide (Grabinski et al., 2015). Contrasting ISH it is, however, difficult to quantify peptide levels at the microscopic level with IHC. Also, IHC requires fixed tissues, whereas snap-frozen fresh tissue is used for ISH. Nevertheless, these histochemical/biochemical approaches have been applied in countless animal experimental studies to explore a possible functional role of neuropeptides in specific neuronal populations.

## Neuropeptide and Small Transmitter Coexistence

In the 1970’s several groups reported that a neuron may release more than one transmitter. These findings were often considered to violate “Dale’s principle,” a rule generally thought to state that a neuron only produces and releases one neurotransmitter. This was subsequently clarified as a misunderstanding (e.g., Eccles, 1986). Several of the early studies on transmitter co-existence focused on *invertebrates*, and only on classic transmitters and not neuropeptides (Kerkut et al., 1967; Brownstein et al., 1974; Hanley et al., 1974; Cottrell, 1976). Since then the analysis of co-transmission in this class of animals has been extremely informative. Thanks to in-depth analyses of the comparatively easily accessible and well-characterized systems in invertebrates using front-line methods, detailed knowledge of the mechanisms underlying co-transmission, and of its functional consequences has been generated (as reviewed in, e.g., Kupfermann, 1991; Bargmann, 1993; Nusbaum et al., 2017; Nassel, 2018). In the present article, the focus is on transmitter coexistence in *mammalian* systems.

In mammals, co-existence of noradrenaline (NA) and serotonin (5-hydroxytryptamine, 5-HT) in the same synaptic vesicle of sympathetic nerves in the pineal gland was reported (Jaim-Etcheverry and Zieher, 1973); but, serotonin presumably originated from pinealocytes and had been translocated into the storage sites with the help of cell and vesicular membrane transporter molecules. At that time, evidence was also presented for a developmental transmitter “switch” from a cholinergic to a noradrenergic transmitter phenotype in sympathetic neurons *in vitro*, with some neurons temporarily expressing both acetylcholine and noradrenaline (Furshpan et al., 1976); later work revealed that this also occurred *in vivo* (Landis and Keefe, 1983). Furthermore, several groups, in particular Burnstock and coworkers, provided evidence that ATP is a transmitter and co-transmitter (Burnstock, 1972), at that time a controversial view (Burnstock, 2012).

This was also the period when attention started to focus on peptides/neuropeptides in the brain. David de Wied and colleagues in the Netherlands studied the effects of pituitary hormones on behavior (de Wied and Bohus, 1966). Guillemin and Schally’s groups discovered that the hypothalamic thyrotropin-releasing hormone is a tripeptide (Boler et al., 1969; Burgus et al., 1970), and several new peptides were isolated from the intestine and brain (Tatemoto and Mutt, 1980; Mutt, 1989). Also substance P was isolated from the intestine (von Euler and Gaddum, 1931), but only after 40 years (!) was it chemically identified as an undecapeptide (Chang and Leeman, 1970; Chang et al., 1971). Last but not least, a very large number of important peptides were isolated from the skin of various frog species by Erspamer et al. (1978). In a visionary review, Burnstock raised the question “Do some nerve cells release more than one transmitter?” with focus on ATP and also mentioning neuropeptides (Burnstock, 1976).

**FIGURE 1:**
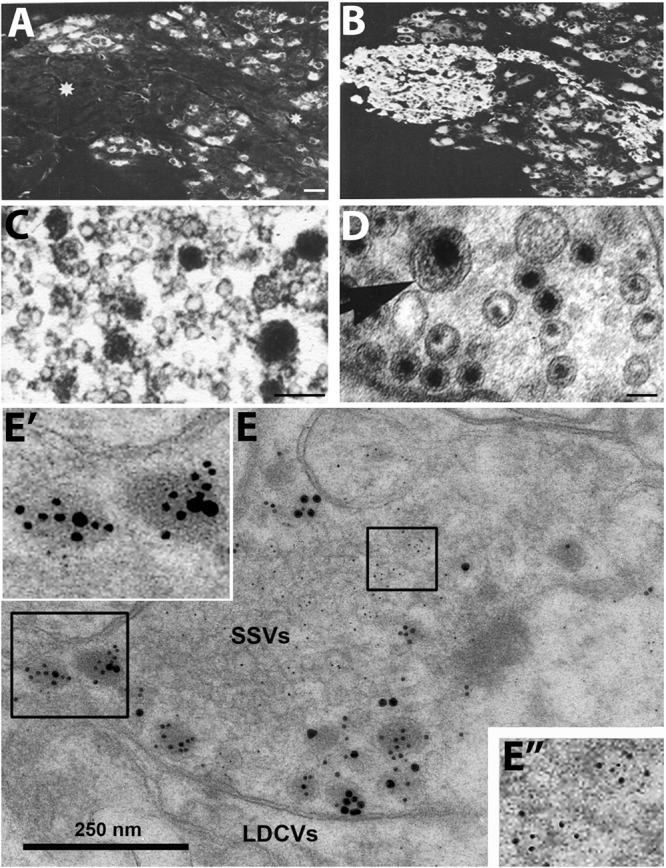
Immunofluorescence micrographs of the guinea-pig inferior mesenteric ganglion **(A,B)** and electron micrographs from different types of nerve endings **(C–E)**. **(A,B)** Two adjacent sections incubated with antibodies to somatostatin **(A)** and the noradrenaline (NA) synthesizing enzyme dopamine ß-hydroxylase (DBH) **(B)**. The majority of the principal ganglion cells are somatostatin-positive, whereas the small intensely fluorescent (SIF) cells (asterisk) lack the peptide. Virtually all ganglion cells and the SIF cells are DBH-positive, i.e., are noradrenergic. **(C–E)** Examples of transmitter storage in nerve endings based on or immunohistochemistry **(C,E)** or potassium permanganate fixation **(D)**. **(D)** In sympathetic nerve endings NA (black precipitate) is stored in both (small) synaptic vesicles and large dense core vesicle (LDCVs) (arrow). Note that content varies between vesicles, both in the synaptic and LDCVs. **(C)** Substance P, a neuropeptide (black precipitate), in a sensory nerve ending in the monkey dorsal horn, is stored exclusively in LDCVs, all synaptic vesicles are empty. **(E)** Peptide and glutamate co-storage and coexistence in the dorsal horn of the rat spinal cord based on immunogold immunohistochemistry. Substance P/CGRP is detected with 10/20 nm gold particles and glutamate with 5 nm gold particles. Note that substance P and CGRP can be stored within the same LDCV (left box, magnified in **E’**). Staining for glutamate is restricted to synaptic vesicles (right box, magnified in **E”**). The results suggest that glutamate, a small molecule transmitter, is *not* stored in LDCVs in sensory nerve endings, and release of peptide and amino acid may be separate events. This contrasts NA (see **D**). Bars: 40 μm, for **(A,B)**; 100 nm for **(C,D)**; 250 nm for **(E)**. **(A,B)** From Hokfelt et al. (1977a). **(C)** From DiFiglia et al. (1982), with permission. **(D,E)** Courtesy of Dr. A. Merighi (cf., Merighi, 2002).

At that time the neuropeptide somatostatin was, surprisingly, localized to peripheral sympathetic neurons (Hokfelt et al., 1977a) already known to signal via NA, the transmitter of sympathetic neurons (von Euler, 1948; Hamberger and Norberg, 1963) (Figures 1A,B). Somatostatin had been discovered as an inhibitor of growth hormone release from the anterior pituitary (Brazeau et al., 1973; Vale et al., 1975; Guillemin, 2008). However, it turned out that somatostatin was not only present, as expected, in neurosecretory nerve endings in the hypothalamic median eminence (Dubois et al., 1974; Hokfelt et al., 1974a; Pelletier et al., 1975), but also in many other brain nuclei (Hokfelt et al., 1974a, 1975a; Brownstein et al., 1975; Dubé et al., 1975; Elde and Parsons, 1975). This indicated roles far beyond that of a hypothalamic hormone controlling pituitary growth hormone release. Then somatostatin was shown to have a depressant action on cortical neurons (Renaud et al., 1975). So somatostatin in noradrenergic neurons was the first example of coexistence of a neuropeptide transmitter with a classic neurotransmitter in mammals (Hokfelt et al., 1977a).

Other early examples of this type of coexistence were vasoactive intestinal polypeptide with acetylcholine (Lundberg et al., 1979), and the neuropeptide Y (NPY) with NA (Lundberg et al., 1982). In the brain substance P was found in 5-HT (serotonin) neurons (Chan-Palay et al., 1978; Hokfelt et al., 1978), and cholecystokinin (CCK) in dopamine neurons (Hokfelt et al., 1980b), followed by many more combinations.

Regarding function, it could be shown, for example, that VIP contributes to the atropine-resistant vasodilation in exocrine glands (Lundberg et al., 1980), that NPY interacts with NA in sympathetic functions (Allen et al., 1982; Lundberg et al., 1982; Ekblad et al., 1984), and that CCK affects dopamine release (Kovacs et al., 1981; Starr, 1982), binding (Fuxe et al., 1981; Murphy and Schuster, 1982) and behavior (Crawley et al., 1984). In an elegant landmark study on a frog sympathetic ganglion Jan and Jan demonstrated that cholinergic presynaptic fibers express and release an LHRH-like peptide that is responsible for the late, slow excitatory post-synaptic potential via ‘volume transmission’ (Jan and Jan, 1982).

Taken together, these findings suggested a new principle: co-transmission - the release of a neuropeptide and a classic (small molecule) transmitter from the same neuron. In fact, the view emerged that neuropeptides *always* ‘co-exist’ with small molecule transmitters. Moreover, many groups, using IHC at the ultrastructural level, found that peptides are stored in large dense core vesicles (LDCVs) (diameter ∼1,000 Å) (Goldsmith and Ganong, 1975; Swaab et al., 1975; Vandesande and Dierickx, 1975; Castel and Hochman, 1976; Dube et al., 1976; Krisch, 1976; Pelletier et al., 1981; Merighi, 2002) (Figures 1C,E), whereas monoamines like NA are present both in synaptic vesicles (diameter ∼500 Å) and LDCVs as shown with potassium permanganate fixation (KMnO4) (Figure 1D) (Richardson, 1966; Hokfelt and Jonsson, 1968). The number of LDCVs in a nerve ending is mostly low compared to synaptic vesicles, indicating a lower content of peptide molecules vs. classic transmitters. However, the affinity at peptide receptors is in the low nanomolar range, allowing efficacious signaling even by low numbers of peptide molecules in the extracellular space.

**FIGURE 2:**
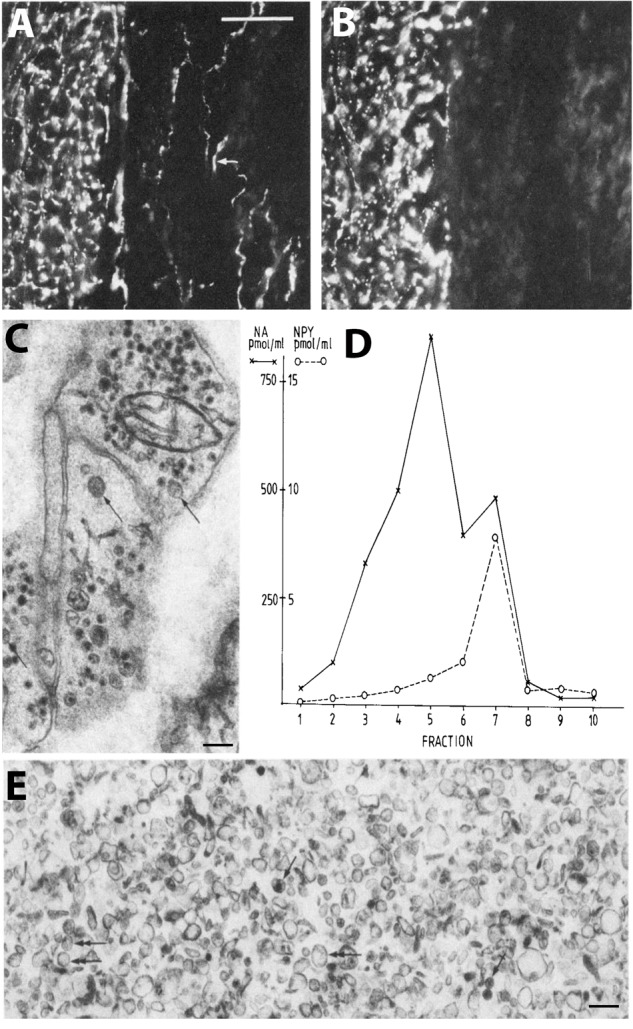
Coexistence and subcellular distribution of neuropeptide Y (NPY) and noradrenaline (NA) in the rat vas deferens. **(A,B)** Immunohistochemical visualization of NPY- **(A)** and tyrosine hydroxylase (TH)-**(B)** positive nerve terminals in adjacent sections. Overlapping, dense NPY and noradrenergic networks are seen in the muscle layer. Note sparse NPY-only positive nerves (arrow) in the subepithelial region, possibly cholinergic nerves. **(C)** Electron microscopic micrograph of several nerve terminal profiles in the muscle layer after potassium permanganate (KMnO4) fixation, showing small synaptic vesicles with a dense core and LDCVs. The dense core indicates presence of NA both in the synaptic and LDCVs (cf. Figure 1D). No profiles without small vesicle with a dense core are seen, suggesting a pure adrenergic innervation of the muscle layer. **(D,E)** Subcellular distribution of NA (x) and NPY (o) in a density gradient of rat vas deferens. There is only one peak for NPY (fraction 7; **E**), whereas there are two peaks for NA (fraction 5 and 7), tentatively representing synaptic vesicles and LDCVs, respectively. Note many LDCVs (arrows), as well as many vesicles of the same size but without dense core (double-headed arrow). The peptide is only present in the heavy fraction (in agreement with Figures 1C,E), whereas NA is present also in the light one (in agreement with Figure 1D). On the abscissa, totally recovered sedimentable substance is given as picomoles per milliliter after centrifugation at 145,000 × *g_max_* for 45 min. On the ordinate, density gradient fractions 1–10 are given, corresponding to the following sucrose molarities: 1 (0.26 M), 2 (0.32 M), 3 (0.47 M), 4 (0.56 M), 5 (0.69 M), 6 (0.74 M), 7 (0.84 M), 8 (0.91 M), 9 (0.98 M), 10 (1.2 M). Recoveries of NA = 70%, of NPY = 65%, and of protein = 87%. Reprinted from Fried et al. (1985), with permission.

It was not clear, whether IHC could exclude that peptides are stored in synaptic vesicles. Pelletier et al. (1981) incubated adjacent, ultrathin sections with antibodies against substance P and 5-HT, respectively, but in both cases *only* LDCVs were stained, not synaptic vesicles. This in spite of the fact that monoamines are (mainly) stored in synaptic vesicles (Figure 1D). Thus, it did not seem possible to visualize the main transmitter (5-HT) in the synaptic vesicles with IHC, contrasting, e.g., the KMnO4 method for NA (Figure 1D). So perhaps IHC also failed to demonstrate *neuropeptides* in synaptic vesicles? Therefore, subcellular fractionation studies were carried out, strongly suggesting lack of peptide in the synaptic vesicle pool but presence of NPY in the fraction with many LDCVs (Figures 2A–E) (Lundberg et al., 1981; Fried et al., 1985)^3^. In contrast to monoaminergic neurons, in sensory glutamatergic neurons the amino acid appears to be exclusively stored in synaptic vesicles (Merighi, 2002) (Figures 1C,E).

**FIGURE 3:**
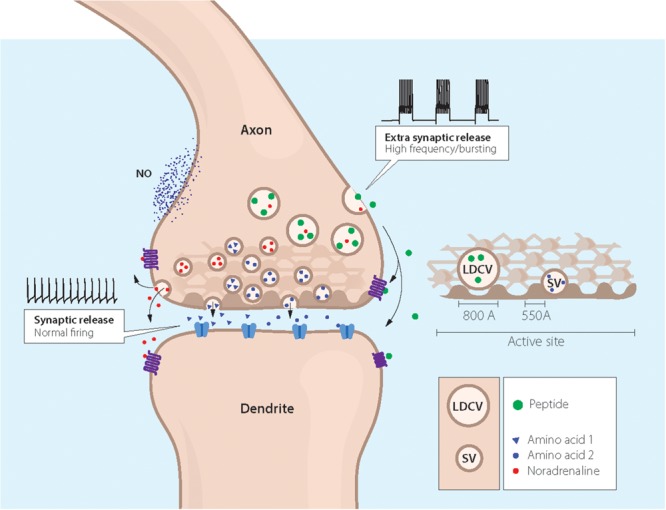
Cartoon showing coexistence of a neuropeptide with classic and ‘unconventional’ neurotransmitters in a nerve ending synapsing on a dendrite. Two types of storage vesicles are shown: synaptic vesicles (diameter 500 Å) storing classic transmitters (e.g., 5-HT, NA, GABA or glutamate), mainly released at synapses; large dense core vesicles (LDCVs) storing neuropeptides and, in amine neurons NA or 5-HT. The peptides are in general released extrasynaptically (“volume transmission”), when neurons fire with high frequency or in bursts. Peptide receptors are essentially extrasynaptic or presynaptic, whereas ligand-gated receptors are mostly localized in the postsynaptic membrane. ‘Gaseous’ (e.g., nitric oxide, NO) and other non-conventional transmitters are not stored in vesicles, but are generated upon demand (Snyder and Ferris, 2000). The presynaptic grid, an egg basket-like structure, originally described by Pfenninger et al. (1969), is indicated in the nerve ending and high-lighted to the right. Note that the LDCV does not fit into the grid and thus cannot attach to the presynaptic membrane for release. In contrast, there is room for the synaptic vesicle. This supports the concept that peptides are mostly not released into the synaptic cleft. Drawing by Mattias Karlen. Modified from Pfenninger et al. (1969),Lundberg and Hokfelt (1983), and Lang et al. (2015).

Furthermore, peptides are in general released when neurons fire at high frequency or in bursts (Lundberg et al., 1980; Andersson et al., 1982; Bondy et al., 1987; Bartfai et al., 1988; De Camilli and Jahn, 1990; Verhage et al., 1991; Consolo et al., 1994; Xia et al., 2009), and often extrasynaptically (Zhu et al., 1986) (Figure 3). The latter was already indicated in a pioneering study on the presynaptic structure of the synapse, showing docking sites for the synaptic vesicles which, however, are not spacious enough to leave room for LDCVs which are twice-the-size (1,000 Å) (Pfenninger et al., 1969) (Figure 3)^4^. This is of course not valid for somato-dendritic release and where true synapses do not exist, nor for the peripheral autonomic nervous system, where there is a considerable distance between the nerve ground plexus (Hillarp, 1949; Falck, 1962) and the smooth muscle cells, as shown with electron microscopy combined with electrophysiology (Merrillees et al., 1963). Furthermore, in the brain, extrasynaptically released neuropeptides may diffuse over long distances, so called volume transmission (Fuxe et al., 2010).

The exocytotic machinery underlying neurotransmitter release has been thoroughly characterized with regard to release of small molecule transmitters stored in synaptic vesicles (De Camilli and Jahn, 1990; Sudhof, 2014). However, the exocytotic neuropeptide release from LDCVs is less well defined. In early studies on synaptosomes it was shown that CCK release from LDCVs is triggered by small elevation of Ca^2+^ concentration in the bulk cytoplasm, whereas glutamate release from the synaptic vesicles requires the higher concentrations produced close to Ca^2+^ channels in the active zone (Verhage et al., 1991). This is in agreement with the localization of the two types of vesicles consistently observed in electron microscopic micrographs of the nerve endings: many synaptic vesicles with some close to the presynaptic membrane, versus a few LDCVs virtually always distant from the synapse (Figure 3).

There is evidence for involvement of SNAREs [soluble *N*-ethyl maleimide (NEM)-sensitive factor attachment protein receptor protein family] (Sudhof, 2014) also in dendritic release from magnocellular dendrites (Schwab et al., 2001; de Kock et al., 2003; Ovsepian and Dolly, 2011). The calcium-dependent activator protein for secretion (CAPS) (Walent et al., 1992) has been identified as a priming factor for exocytosis of LDCVs (Stevens and Rettig, 2009; James and Martin, 2013). Thus CAPS2, but not CAPS1, is required for LDCV exocytosis as shown in cerebellar granule cells and hippocampal interneurons (Sadakata et al., 2004; Shinoda et al., 2011).

Taken together, these early findings suggested that neuropeptides were not the main neuronal messengers. Moreover, when neuropeptides are released, the fast small molecule transmitters are already active in the synaptic cleft – i.e., no peptide release without release of classic transmitters. The discovery of coexistence and co-transmission was summarized in several books/reviews (Burnstock, 1978; Hokfelt et al., 1980a, 1986, 1987a; Cuello, 1982; Chan-Palay and Palay, 1984; Jaim-Etcheverry, 1994; Merighi, 2002), and since then further efforts have been made to understand co-signaling involving neuropeptides, including co-release of both an excitatory and an inhibitory neuropeptide. For an up-to-date overview of many aspects on neuropeptide signaling (see e.g., Salio et al., 2006; van den Pol, 2012; Ludwig et al., 2016).

More recently it has become clear that coexistence of small molecule transmitters, encompassing various combinations of GABA, glycine, glutamate, dopamine and acetylcholine (e.g., Guiterrez, 2009; Hnasko and Edwards, 2012; Trudeau et al., 2014) (Figure 3). For example, coexistence of GABA and glycine was first reported in the cerebellum (Ottersen et al., 1988), and then in the spinal cord (Todd and Sullivan, 1990; Ornung et al., 1994), where evidence for GABA-glycine co-transmission was obtained in the dorsal horn, and possible co-release from the same synaptic vesicles (Jonas et al., 1998) (Figure 3). Moreover, mesencephalic dopamine neurons can also release glutamate (Hnasko et al., 2010) and GABA (Tritsch et al., 2012), whereby GABA is *not* synthesized via the classic enzyme glutamate decarboxylase (GAD) but via aldehyde dehydrogenase 1a1 (Kim et al., 2015).

Thus, the number and combinations of transmitters present in a nerve ending (and/or dendrites) virtually seem endless, and it is difficult to define rules according to which neurotransmitters co-exist and are involved in co-transmission, as is discussed further in this Frontiers special topic. Furthermore, neurotransmitter switching, the gain of one and loss of another transmitter in the same, mammalian neuron, can occur not only during development but also in adult animals (Spitzer, 2017).

There is an increasing interest in neuropeptide/neurotrans-mitter coexistence and a need to explore transcriptional changes in defined healthy and diseased brain circuitries (Akil et al., 2010). In fact, there are many interesting results from *animal* disease models, suggesting involvement of neuropeptides and neuropeptide coexistence in patho-physiological processes with potential therapeutic implications. However, information on the significance of transmitter and neuropeptide *coexistence* in the normal and diseased *human* nervous system is limited. In this article, the focus is on galanin co-existing in noradrenergic neurons in the LC, and on galanin receptor expression in *postmortem* brains from normal subjects and depressed patients who committed suicide (Le Maitre et al., 2013; Barde et al., 2016). This is in line with previous extensive work carried out on *postmortem* brains from depressed humans, showing changes in transcripts related to neurotransmitters/neuropeptides and their receptors and to transporters, growth factors in nerve cells, and in glia, in cortical, limbic, hypothalamic and lower brain stem regions (Evans et al., 2004; Iwamoto et al., 2004; Aston et al., 2005; Choudary et al., 2005; Kang et al., 2007; Anisman et al., 2008; Kozicz et al., 2008; Tochigi et al., 2008; Klempan et al., 2009; Sequeira et al., 2009, 2012; Sibille et al., 2009; Poulter et al., 2010; Bernard et al., 2011; Bloem et al., 2012; Kerman et al., 2012; Zhurov et al., 2012; Labonte et al., 2013, 2017; Li et al., 2013; Du et al., 2014; Lopez et al., 2014a,b; Hayley et al., 2015; Maheu et al., 2015; Torres-Platas et al., 2016; Roy et al., 2017).

## Galanin

**FIGURE 4:**
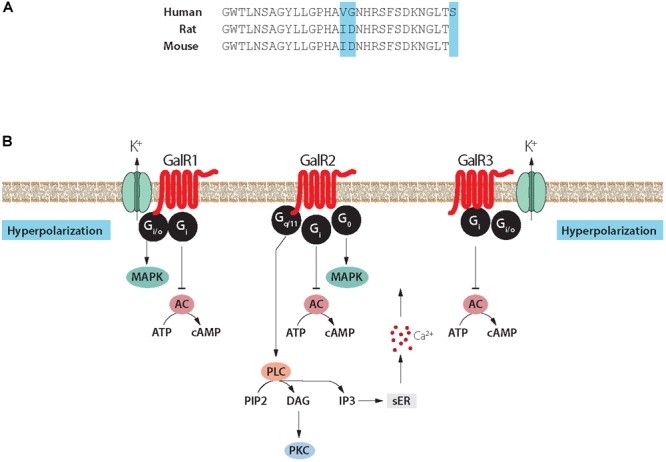
**(A)** Structure of galanin in three species. Galanin is composed of 29 amino acids in most species, except humans (30 amino acids). Note conservation of N-terminal portion. **(B)** Signaling pathways of galanin receptor subtypes. Galanin, via GalR1 and GalR3, opens potassium channels leading to membrane hyperpolarization. Galanin can via GalR2 activate PLC resulting in generation of IP3, release of Ca^2+^ from the smooth endoplasmic reticulum, opening of Ca^2+^ channels and eventually transmitter release. AC, adenylate cyclase; cAMP, 3′, 5′-cyclic adenosine monophosphate; DAG, diacylglycerol; K^+^, G-protein-regulated inwardly rectifying potassium channel; sER, smooth endoplasmic reticulum; IP_3_, inositol triphosphate; PIP_2_, phosphatidylinositol bisphosphate; PKC, protein kinase C; PLC, phospholipase C. Modified from Iismaa and Shine (1999) and Lang et al. (2015). Drawing by Mattias Karlén.

Galanin was originally isolated from porcine intestine as a 29-amino acid (30 in humans) neuropeptide (Tatemoto et al., 1983; Schmidt et al., 1991) (Figure 4A) with a wide distribution in the rat brain as shown with RIA (Skofitsch and Jacobowitz, 1986), IHC (Rokaeus et al., 1984; Melander et al., 1985, 1986b,c,d; Skofitsch and Jacobowitz, 1985; Merchenthaler et al., 1993), and ISH (Gundlach et al., 1990b; Jacobowitz and Skofitsch, 1991; Jacobowitz et al., 2004). The distribution of galanin in the mouse brain is similar to that in rat, both with regard to galanin peptide (Perez et al., 2001) and to its mRNA (Cheung et al., 2001; Lein et al., 2007). The galanin system has also been characterized in the monkey brain (Melander and Staines, 1986; Kordower and Mufson, 1990; Walker et al., 1991) (for human brain, see below).

For many years galanin was considered as the sole endogenous ligand for GalR1-3 but more recently additional ligands were described (Lang et al., 2015)^5^. Currently, three galanin receptors, GalR1-3, have been cloned, all three belonging to the family of seven transmembrane-spanning GPCRs, with different transduction mechanisms, with GalR1 and -R3 having distinct similarities (Habert-Ortoli et al., 1994; Fathi et al., 1997; Howard et al., 1997; Wang et al., 1997; Ahmad et al., 1998; Smith et al., 1998; Iismaa and Shine, 1999; Branchek et al., 2000; Lang et al., 2007, 2015) (Figure 4B). The three galanin receptors are present in most parts of the rat brain, but could not be detected e.g., in dorsal cortical areas and the hippocampal formation (HiFo) in early autoradiographic ligand binding studies (Skofitsch et al., 1986; Melander et al., 1986a, 1988).

Galanin receptors have also been mapped in the mouse brain using 125I-galanin binding autoradiography (Jungnickel and Gundlach, 2005). A direct comparison with results in rat in the study by, e.g., O’Donnell et al. (2003) reveals an overall similar distribution but with some remarkable, apparently qualitative species differences. Thus, mouse shows, i.a., a strong signal in two important regions, the striatum and the cerebellum (Jungnickel and Gundlach, 2005) which both lack binding in the rat (Skofitsch et al., 1986; Melander et al., 1988; O’Donnell et al., 2003). To our knowledge, no attempts have been made to identify the cellular localization and origin of, e.g., the structures binding galanin in the mouse striatum.

The cloning of the receptors allowed localization with ISH and qPCR, which revealed that the transcripts for GalR1 and GalR2 are widely distributed in the rat brain, primarily in the brain stem and in ventral cortical areas (Landry et al., 1998; Mitchell et al., 1999; O’Donnell et al., 1999, 2003; Burazin et al., 2000; Waters and Krause, 2000; Mennicken et al., 2002). However, the GalR2 transcript is transiently highly expressed in neocortex during the first week after birth (Burazin et al., 2000). The distribution of GalR3 is limited (Mennicken et al., 2002). Only the GalR1 transcript has been mapped with ISH in the mouse brain (Hohmann et al., 2003; Lein et al., 2007). Thus, The Allen Brain Atlas (Lein et al., 2007) lacks results on GalR2 or GalR3, suggesting that they are expressed at low levels. This is also supported by the demonstration that the 125I-galanin binding sites are absent in a GalR1 knock-out mouse (Jungnickel and Gundlach, 2005). Taken together, these results suggest that GalR1 is the predominant receptor in the mouse brain, and that distinct species differences exist between mouse and rat.

GalR3 has emerged as a complex receptor (Lang et al., 2015), not present in all mammals (Liu et al., 2010). Its signaling properties are still not well defined, even though GalR3-transfected cell lines have now been generated (Lu et al., 2005b; Robinson et al., 2013). However, these cells could so far not be used for stable signaling experiments (see Lang et al., 2015). Still, GalR3 presumably acts via a PTX sensitive G_i/o_-type G protein which in turn regulates inwardly rectifying K^+^ channels (Smith et al., 1998), as do GalR1 receptors (Smith et al., 1998) (Figure 4B). This lack of knowledge contrasts the substantial information about GalR1 and GalR2 (see Lang et al., 2015). The cloning of the receptors was useful, also because it has been difficult to raise specific antibodies to GalR1-3 (Lu and Bartfai, 2009; Brunner et al., 2018). A similar situation exists for other GPCRs (Michel et al., 2009). Detailed tables on the distribution of galanin and GalR1-3 in rodent brain are found in O’Donnell et al. (1999, 2003), Burazin et al. (2000), Hohmann et al. (2003), and Jungnickel and Gundlach (2005).

Early research on galanin was initiated because of its strong reaction to nerve injury. Transection of the sciatic nerve in rat causes an >100-fold increase in galanin synthesis (mRNA and peptide levels) in the corresponding somata of DRG somata (Hokfelt et al., 1987b). Upregulation could also be detected in the brain after various types of injury/manipulations (Cortes et al., 1990a,b; Villar et al., 1990; Agoston et al., 1994; Palkovits, 1995). In fact, galanin meets the criteria of a neurotransmitter/-modulator, but also has trophic functions, as shown both in brain and the peripheral nervous system (Hobson et al., 2010). Galanin has, in fact, many characteristics similar to the brain-derived neurotrophic factor (BDNF), including storage in, and exocytotic release from LDCVs and both transmitter and trophic functions (Barde, 1994). For example, galanin affects spine density (Sciolino et al., 2015), and it is well-known that BDNF influences dendritic morphology (Bennett and Lagopoulos, 2014). Thus, trophic functions of galanin are potentially interesting but will not be discussed here.

**FIGURE 5:**
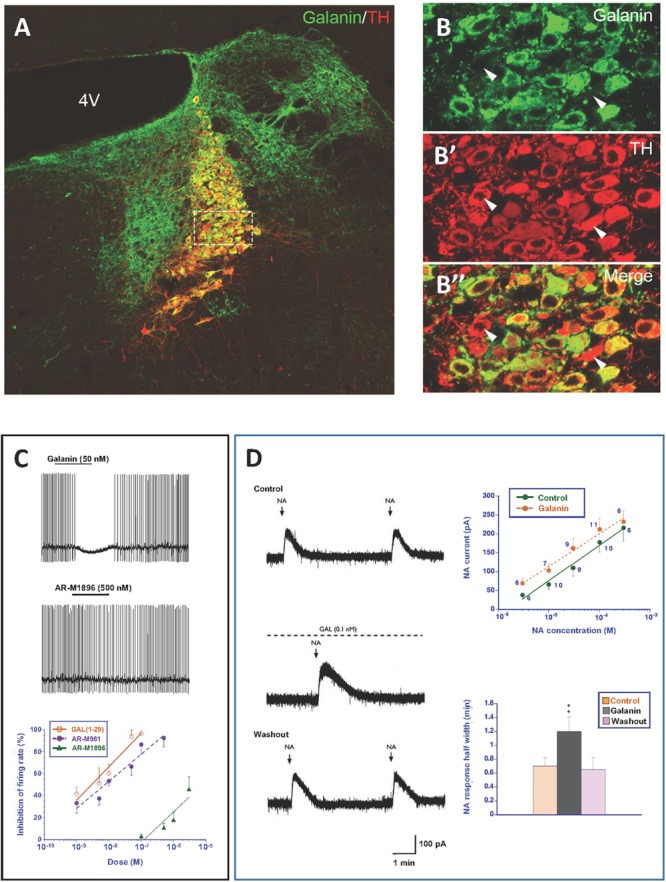
**(A–B”)** Immunofluorescence micrographs of the dorsal pontine periventricular region of mouse after double-staining of a section with antibodies to galanin (green) and tyrosine hydroxylase (TH) (red), the rate-limiting enzyme for catecholamine synthesis and thus a marker for NA neurons. Note that both antibodies stain neurons in the locus coerulus (LC) **(B,B’)**, whereby many (yellow, **B”**), but not all TH-positive neurons express galanin [arrowheads point to TH-only neurons (red), apparently lacking galanin] **(B’)**. Galanin is also present in many structures outside the LC. Colchicine treated animal. Courtesy Joanne Bakker and Mingdong Zhang. Bar for **(A)** 200 μm, for **(B–B”)** 20 μm. **(C)** Effect of galanin and the GalR2 agonist AR-M1896 on LC neurons (upper two traces), and the dose–response curves of galanin (red), the AR-M1896 (green) and the mixed GalR1-GalR2 M961 agonist (magenta) (lower trace). Note strong hyperpolarization of galanin and a less strong effect of M961, whereas that AR-M1896 hardly causes any effect at all. From Ma et al. (2001). (**D**, left panel) Effect of galanin on the response of LC neurons to NA. NA (applied from a pipette at the arrowhead) induces a persistent outward current (upper trace). When galanin (0.1 nM) is present, the NA-induced outward current is enhanced, and the duration is prolonged (middle trace). After wash out of galanin, the amplitude and duration of the NA response was similar to that seen before galanin administration (lower trace). (**D**, right panel) Effect of galanin on dose-response (upper figure) and duration (lower figure) of NA. The NA dose-response curve is shifted to the left, when galanin (0.1 nM) is present (upper figure). The duration of the NA-induced current is increased in the presence of galanin (lower figure). ^∗∗^*P* < 0.01. From Xu et al. (2001) with permission.

A further early finding in the rat was the coexistence (Figures 5A,B”) of galanin (Figure 5B) in both noradrenergic neurons in the LC (Figure 5B’) (Rokaeus et al., 1984; Skofitsch and Jacobowitz, 1985; Melander et al., 1986b,c; Holets et al., 1988; Moore and Gustafson, 1989) and in serotonergic neurons in the dorsal raphe nucleus (DRN) (Melander et al., 1986c; Fuxe et al., 1990; Priestley et al., 1993; Xu and Hokfelt, 1997), two systems associated with mood-related behavior. The LC neurons also express transcripts for both GalR1 and -R2 (O’Donnell et al., 1999; Burazin et al., 2000).

Thereafter galanin biology has since the early 1990’s been regularly summarized in books/journal from meetings (Hökfelt et al., 1991, 1998; Hökfelt and Crawley, 2005; Hokfelt, 2010; Hokfelt and Tatemoto, 2010); and in peer-reviewed articles focusing on the nervous system (only such published after 2004, and not included in the books/journals cited above, are listed here) (Lundstrom et al., 2005; Holmes and Picciotto, 2006; Karlsson and Holmes, 2006; Ogren et al., 2006, 2007, 2010; Robinson et al., 2006; Walton et al., 2006; Wrenn and Holmes, 2006; Lu et al., 2007; Tortorella et al., 2007; Picciotto, 2008; Robinson and Brewer, 2008; Butzkueven and Gundlach, 2010; Picciotto et al., 2010; Webling et al., 2012; Diaz-Cabiale et al., 2014; Freimann et al., 2015; Weinshenker and Holmes, 2016; Millon et al., 2017a; Genders et al., 2018a); and in some major comprehensive reviews (Lang et al., 2007, 2015).

## Galanin Inhibits Rat Locus Coeruleus Neurons

Locus coeruleus is a small, compact bilateral nucleus in the pons located in the gray matter close to the lateral aspect of the 4^th^ ventricle (Maeda, 2000). Dahlstrom and Fuxe first reported that NA is a transmitter in the rat LC, a.k.a. the A6 group (Dahlstrom and Fuxe, 1964). They used the formaldehyde, or Falck-Hillarp, fluorescence method that allows microscopic visualization of catecholamines and serotonin in tissue sections (Carlsson et al., 1962; Falck, 1962; Falck et al., 1962).

In the rat, the LC contains 2,800–3,600 neurons (both sides) (with an additional 260 neurons in the subcoeruleus area, the vast majority of which are noradrenergic with wide projections to virtually all parts of the central nervous system (Ungerstedt, 1971; Descarries and Saucier, 1972; Swanson and Hartman, 1975; Swanson, 1976; Morrison et al., 1978; Moore and Bloom, 1979; Goldman and Coleman, 1981; Foote et al., 1983; Aston-Jones et al., 1995). NA nerve terminals are also extensively present in primate cortex (Lewis et al., 1986).

When explored with electrophysiological methods galanin has effects on the membrane potential of several neuron systems (see Xu et al., 2005). Galanin hyperpolarizes noradrenergic LC neurons in a slice preparation (Seutin et al., 1989; Sevcik et al., 1993; Pieribone et al., 1995), mediated via GalR1 (Ma et al., 2001) (Figure 5C). However, the GalR2 (R3) agonist ARM-1986 (Liu et al., 2001; Lu et al., 2005b) does not cause any effect on the membrane potential (Ma et al., 2001) (Figure 5C). GalR2 may instead have a presynaptic role in the projection areas of LC neurons, perhaps mainly acting as an autoreceptor (Ma et al., 2001). In agreement, galanin is present in noradrenergic [dopamine ß-hydroxylase (DBH)]-positive nerve terminals in cortex and the hippocampus (Melander et al., 1986d; Xu et al., 1998). Galanin activation of GalR1, but not -R2 or R3, has been shown also in other studies on the rat and mouse LC (Hawes et al., 2005; Mitsukawa et al., 2009). In addition to this direct effect, galanin at *low* concentrations (10^-9^M) enhances the autoinhibitory effect of NA on LC neurons via alpha-2A receptors (Xu et al., 2001) (Figure 5D). This may in fact be the primary action of galanin in controlling the firing of LC neurons. Thus, galanin can via different autoinhibitory mechanisms exert a two-step inhibition on LC neurons, at low concentrations enhancing the inhibitory alpha-2A receptor effect.

Autoinhibition of LC neurons, mediated by NA via alpha-2A receptors, was early discovered by Svensson et al. (1975) and Aghajanian et al. (1977). It is assumed that autoinhibition, both at NA and serotonin neurons, at least in part, is responsible for the delayed onset of the clinical effect of monoamine reuptake inhibitors (Artigas et al., 1996; Mongeau et al., 1997; Millan, 2006). Autoinhibition via NA in LC was originally suggested to be a consequence of the release from collaterals (Aghajanian et al., 1977). There is, however, evidence that NA can be released from soma/dendrites (Pudovkina et al., 2001; Pudovkina and Westerink, 2005), and more recently release was shown to occur from individual vesicles by combined measurements using amperometry and patch clamp methodologies (Huang et al., 2007). This is in agreement with electron microscopic analysis, showing synaptic vesicles with a dense core in LC dendrites (Shimizu et al., 1979). Thus, collaterals are not necessarily the only structure involved in the autoinhibition.

**FIGURE 6:**
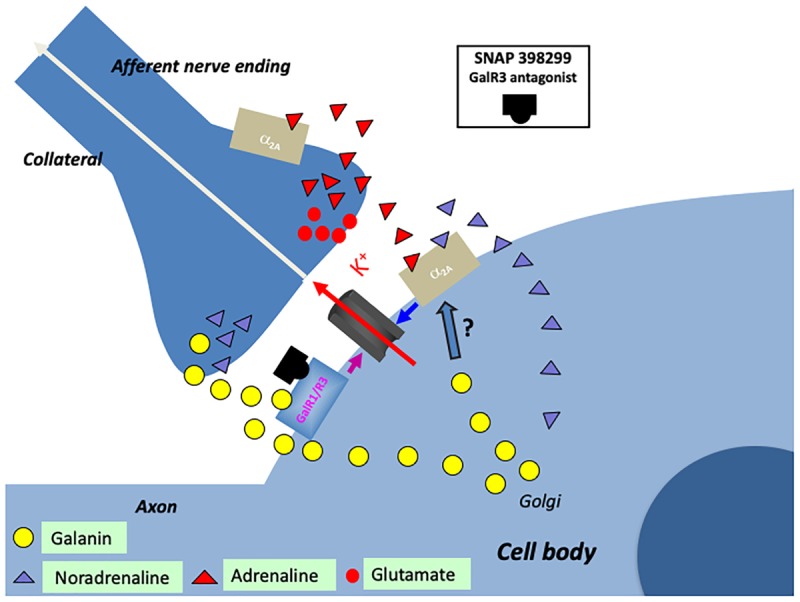
Cartoon showing several transmitters and signaling pathways in the locus coeruleus (LC) (part of a cell body with initial axon and an afferent nerve ending and a possible axon collateral). A noradrenergic LC neuron co-expresses galanin (yellow LDCVs) originating in the Golgi complex. The peptide in the LDCVs is, after transport to the somatic and dendritic cell membrane, released by exocytosis. Galanin then acts on inhibitory autoreceptors (GalR1/R3), opening potassium channels, in this way attenuating noradrenaline (NA) release in the forebrain. Galanin at low concentrations enhance the alpha2A mediated inhibition of the LC neuron (by an unknown mechanism). Galanin could also be released from collaterals. The GalR3 antagonist (SNAP 398299) may exert an antidepressive action by disinhibiting the LC neuron and restituting forebrain NA levels. With regard to small transmitters, NA (purple triangles) can be released from soma-dendrites and collaterals, acting on somato-dendritic, postsynaptic and presynaptic alpha2A receptors. The afferent nerve ending originates from C1 neurons which are glutamatergic (red dots) and co-release adrenaline (red triangles). Also adrenaline can act on the alpha2A receptors. The basis for this cartoon is animal experiments, and in the case of the galanin system, results from human postmortem brains are also incorporated.

There is another source of catecholamine input to the LC neurons originating from one of the three C neuron groups in the medulla oblongata: adrenaline (epinephrine) containing afferents (Figure 6) (Hokfelt et al., 1974b, 1984; Howe et al., 1980; Armstrong et al., 1982), which synapse on LC dendrites (Milner et al., 1989). This was supported by early tracing experiments, although at that time no transmitter histochemical identification was performed (Cedarbaum and Aghajanian, 1978). One likely origin is C1 neurons, since they display a high degree of collateralization, including inputs to the LC (Figure 6) (Haselton and Guyenet, 1990).

Early studies suggested that the adrenaline (Cedarbaum and Aghajanian, 1976) and the C1 neurons (Aston-Jones et al., 1991) inhibit LC neurons. However, the more recent discovery that the C1 neurons are glutamatergic together with optogentic analysis demonstrated excitation as the primary effect (Figure 6) (Abbott et al., 2012). Released adrenaline may act as a modulator not only on postsynaptic but also presynaptic (Li et al., 1995) alpha-2A receptors, which will, respectively, directly and indirectly, dampen LC neuron activity (Figure 6) (Guyenet et al., 2013).

Taken together, galanin prevents overexcitation of LC, but is only one of several molecules performing this task (Aston-Jones et al., 1991; Singewald and Philippu, 1998; Van Bockstaele, 1998; Berridge and Waterhouse, 2003; Van Bockstaele and Valentino, 2013). This comprehensive network is perhaps a sign of how important it is to balance the activity of the noradrenergic LC neurons, which are involved in the control of many bodily functions (see below).

Kehr and colleagues have analyzed the effect of intracerebroventricularly administered galanin in freely moving rats and mice, monitoring several neurotransmitters using *in vivo* microdialysis (Ungerstedt, 1984) and a sensitive HPLC method. Their studies indicate that galanin reduces basal and desipramine-induced extracellular NA levels (Yoshitake et al., 2003, 2004). This effect is assumed to be exerted via GalR1 at the noradrenergic cell bodies/dendrites in the LC.

### Galanin and Dendritic Release

Studies on the hypothalamic magnocellular hormones vasopressin and oxytocin have provided compelling evidence that these two peptides are not only released from nerve endings in the posterior pituitary but also, independently, from dendrites in the paraventricular and supraoptic nuclei (Morris et al., 1998; Landgraf and Neumann, 2004; Ludwig and Leng, 2006; Kennedy and Ehlers, 2011; Ovsepian and Dolly, 2011; Ludwig et al., 2016). There is evidence for involvement of SNAREs [soluble *N*-ethyl maleimide (NEM)-sensitive factor attachment protein receptor protein family] (Sudhof, 2014) in release from magnocellular dendrites (Schwab et al., 2001; de Kock et al., 2003; Ovsepian and Dolly, 2011). Results from studies on CAPS2-dependant neuropeptide release from soma of dorsal root ganglion neurons (Bost et al., 2017; Shaib et al., 2018) may also be relevant for dendritic/somatic release in the brain. Galanin may be released from soma and dendrites in the LC (Pieribone et al., 1995; Vila-Porcile et al., 2009) (Figure 6). Therefore, it has been hypothesized that stress-induced firing increases galanin release from nerve terminals in the forebrain and dendrites-soma of LC neurons. This could lead to activation of GalR1 autoreceptors and inhibition of firing of LC neurons, a possible mechanism involved in resilience and development of depression-like behavior in animals (Sciolino et al., 2015) (see below).

### Other Co-transmitters in the LC

Neuropeptide Y is expressed in LC neurons in rat (Everitt et al., 1984; Chronwall et al., 1985; Yamazoe et al., 1985; Holets et al., 1988) and human (Chan-Palay et al., 1990). Recently it has been shown in mice that dopamine is co-released with NA in the hippocampus (Kempadoo et al., 2016; Takeuchi et al., 2016) and the paraventriculer *thalamic* nucleus (Beas et al., 2018) and is involved in memory consolidation and control of stress responsitivity, respectively.

## Galanin and Depression-Like Behavior in Rodents

Galanin influences mood-related behavior in a region-specific way (Bing et al., 1993; Moller et al., 1999). Moreover, results from a number of rat experimental models suggest that galanin can be both prodepressive/anxiogenic and antidepressive (Fuxe et al., 1990, 1991, 1998; Weiss et al., 1998, 2005; Bellido et al., 2002; Khoshbouei et al., 2002; Barrera et al., 2005; Sergeyev et al., 2005; Lu et al., 2005a, 2007, 2008; Holmes and Picciotto, 2006; Karlsson and Holmes, 2006; Ogren et al., 2006; Kuteeva et al., 2008, 2010; Kozlovsky et al., 2009; Picciotto et al., 2010; Le Maitre et al., 2011; Sciolino et al., 2012, 2015; Weinshenker and Holmes, 2016).

In many of the early studies listed above on depressive-like behavior the receptor involved was not identified, or the site of action was not defined experimentally, but there was a general consensus that it is GalR1 that mediates the depressive behavior and that GalR2 may be prodepressive (summarized in Mitsukawa et al., 2009; Kuteeva et al., 2010; Hoyer and Bartfai, 2012; Webling et al., 2012; Freimann et al., 2015).

Two recent studies support involvement of GalR1, and suggest the ventral periaqueductal gray as one likely site of action. Using a rat model of depression based on chronic mild stresses (CMS) (Willner et al., 1987; Moreau et al., 1992), behavior was evaluated in the open field test, the forced swim test (FST), and by monitoring sucrose consumption (Wang et al., 2016). Transcript levels of galanin and GalR1-3 in various, laser-dissected brain regions, including the hippocampal formation (HiFo), vPAG, the DRN and the LC were analyzed with quantitative real time PCR (qPCR) (Wang et al., 2016). Only GalR1 mRNA levels were significantly changed (increased), in a single region, the vPAG. Moreover, after knocking down GalR1 in the vPAG using siRNA, the depressive behavioral phenotypic parameters were similar to unstressed controls. This result suggested that the depression-like behavior in rats exposed to CMS is likely related to an elevated expression of GalR1 in the vPAG. The phenotype of the GalR1-positive neurons was not identified, despite comparing their distribution with serotonin, glutamate (vesicular glutamate transporter type 2, VGLUT2) and GABA (glutamic acid decarboxylase, GAD) neurons (Wang et al., 2016).

In another study, the galanin system was monitored with qPCR, ISH and RIA methodologies following mild blast-induced traumatic brain injury (mbTBI) (Kawa et al., 2016). Significant increases in galanin peptide and transcript were observed in the LC, at 1 day with qPCR, at 3 days with RIA and from 2 h to 7 days with ISH. The increases thus remained for 7 days (ISH) (the longest period studied). With regard to galanin receptors, GalR1 mRNA was significantly increased in vPAG at 1 and 7 days, likely in the same neuron population as seen in the CMS model (Wang et al., 2016). These findings suggest a long-lasting role for the galanin system in the endogenous response to mbTBI. Again, the phenotype of these GalR1-positive neurons was not identified. Nevertheless, in both cases stress, and possibly depression-like behavior, are associated with increased levels of GalR1 transcript in the vPAG. Interestingly, the galanin system has also been shown to modulate stress-related responses related to mild TBI in a model of postraumatic stress disorder (PTSD) (Kozlovsky et al., 2009).

The robust and lasting effect of mbTBI on the expression of galanin (at least 7 days) not only in LC but also in 5-HT neurons (Kawa et al., 2016) is more sustained than the transient increase in tyrosine hydroxylase (TH) (3 days) and tryptophan hydroxylase 2 (1 day) seen in the same *mbTBI* model (Kawa et al., 2015). Thus, in mbTBI the coexisting peptide may have a more long-lasting and important effect than the small molecule transmitter.

In another study, i.p. injection (a stress by itself) and swim stress increased both galanin and TH mRNA levels in the LC, but not TPH2 or galanin transcripts in the DRN (Kuteeva et al., 2008), indicating that the serotonergic system is less sensitive to stress than the noradrenergic system. This has also been shown in other studies employing different types of stress (e.g., Wilkinson and Jacobs, 1988; Jordan et al., 1994; Kuteeva et al., 2008, 2010). The long lasting effects of stress can also be gauged against the fact that peptides can exert effects over long periods of time (Herbert, 1993; van den Pol, 2012). One example is a study on the lamprey locomotor network that revealed that a 10-min administration of substance P causes a long-lasting (>24 h) modulation of the frequency and regularity of NMDA-evoked locomotor bursts (Parker and Grillner, 1999).

**FIGURE 7:**
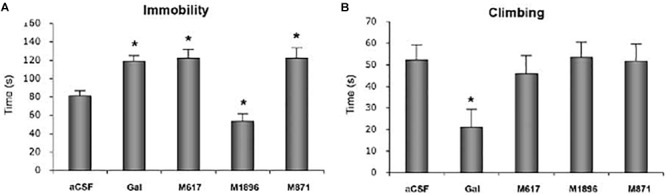
Time of immobility **(A)** and climbing **(B)** in the forced swim test (FST). Rats received i.c.v. infusion of aCSF, galanin (Gal), the GalR1 receptor agonist M617, the GalR2(R3) agonist AR-M1896 or the GalR2 antagonist M871 (M871) 20 min prior to a 5 min test. Data presented as mean ^±^SEM. significant difference from the control swim group; ^∗^one-way ANOVA, Fisher’s PLSD. Galanin, the GalR1 agonist and the GalR2 antagonist increase the immobility time versus a decrease after the GalR2(3) agonist. From Kuteeva et al. (2008), with permission.

GalR2 may also be involved, but here an opposite effect has been recorded, i.e., galanin actions via this receptor are antidepressive (Gottsch et al., 2005; Lu et al., 2005a, 2007, 2008; Kuteeva et al., 2008, 2010; Kinney et al., 2009; Le Maitre et al., 2011; Saar et al., 2013a,b; Kawa et al., 2016), in some cases associated with the vPAG. For example, in a neuropharmacological study (Kuteeva et al., 2008) the time of immobility (Figure 7A) and climbing (Figure 7B) were recorded in the FST. Galanin, the GalR1 receptor agonist M617, the GalR2(R3) agonist AR-M1896, the GalR2 antagonist M871 or aCSF were infused intracerebroventricularly. Galanin significantly increased immobility time, as did the GalR1 receptor agonist M617 (Figure 7A). In contrast, the GalR2(R3) agonist AR-M1896 decreased immobility, similar to fluoxetine, whereas the GalR2 antagonist M871 *increased* the time of immobility. Together these results support the view of GalR1 being pro- and GalR2 antidepressive. Moreover, the antidepressive effect of the GalR2 antagonist suggests there is an *in vivo*, tonic activation of this receptor under forced swimming (stress). This provides further evidence that galanin is released *in vivo* under stressful conditions. More recently it has been shown that an anxiolytic-/antidepressive effect of galanin injected directly into the DRN is mediated via GalR2 (Silote et al., 2013; de Souza et al., 2018).

Taken together, galanin receptors GalR1 and GalR2 play a differential role in regulation of depression-like behavior. Thus, galanin exerts a prodepressive effect, presumably via GalR1, while stimulation of GalR2 has an antidepressant-like effect.

While little interest has been paid to GalR3 in relation to mood, possibly due to its low expression in the rat (Mennicken et al., 2002) and mouse (Lein et al., 2007) brain, a GalR3 knockout mouse exhibits an anxiety-like phenotype (Brunner et al., 2014).

Following early studies on intra-membrane receptor-receptor interactions (Fuxe and Agnati, 1985), receptor di- and heteromerization have become a recognized mechanism for signaling through GPCRs (Bouvier, 2001; Devi, 2001; Agnati et al., 2003). Recent studies reveal that galanin receptor heteromers exist, introducing a further degree of complexity in interpreting galaninergic signaling in the brain (Fuxe et al., 2012), and in relation to mood control. Thus, in addition to GalR1 and 5-HT1A receptor heterodimers (Borroto-Escuela et al., 2010), the galanin (1–15) fragment alone induces strong depression- and anxiogenic-related effects and may regulate mood via binding to GalR1 and GalR2 heterocomplexes (Millon et al., 2014, 2017a,b). Interestingly, galanin (1-15) causes a dose-dependent hyperpolarization of a population of hippocampal CA3 neurons (Xu et al., 1999), and after iodination it binds to other regions including the dorsal hippocampus, as shown in autoradiographic studies (Hedlund et al., 1992). Taken together these results provide evidence for a functional role of galanin (1–15), perhaps unexpected in view of results showing a high affinity of the N-terminal galanin *(1-16)* fragment to galanin binding sites in the brain (Fisone et al., 1989).

## Galanin and Depression-Like Behavior in Rodents – Lc

The LC and NA have since the 1960’s been a focus of clinical and preclinical monoamine research, because of their involvement in stress, mood control and treatment of mood disorders (Bunney and Davis, 1965; Schildkraut, 1965; Weiss et al., 1981, 1994; Svensson, 1987; Simson and Weiss, 1988; Page and Valentino, 1994; Schatzberg and Schildkraut, 1995; Aston-Jones et al., 1996; Bremner et al., 1996a,b; Harro and Oreland, 2001; Charney, 2004; Millan, 2006; Samuels and Szabadi, 2008; Seki et al., 2018).

There is a strong relationship between stress and the LC: stress increases NA turnover, as well as tyrosine hydroxylase activity and transcription in the LC (Korf et al., 1973; Zigmond et al., 1974; Abercrombie and Jacobs, 1987; Komori et al., 1990; Smith et al., 1991; Melia et al., 1992; Aston-Jones et al., 1996; Rusnak et al., 1998; Chang et al., 2000; McDevitt et al., 2009; Ong et al., 2014; Kawa et al., 2015). Moreover, stress activation of LC neurons results in release of NA in the forebrain (Abercrombie et al., 1988; Jordan et al., 1994; Vahabzadeh and Fillenz, 1994; Ihalainen et al., 1999; Yoshitake et al., 2004) and cortical EEG activation, i.e., arousal (Page et al., 1993). Here CRF (Vale et al., 1981) is an important mediator of the stress-induced LC activation (Valentino and Van Bockstaele, 2015).

Also galanin expression is upregulated in LC neurons in response to stress/exercise (Holmes et al., 1995; Sweerts et al., 1999; O’Neal et al., 2001; Sciolino et al., 2012; Weinshenker and Holmes, 2016), establishing a relation between stress, NA and galanin in LC. Similarly, a single dose of the monoamine-depleting drug reserpine (Pletscher et al., 1955; Carlsson, 1975) causes an increase in galanin mRNA levels in LC neurons (Austin et al., 1990; Gundlach et al., 1990a). The same treatment results in a complete depletion of galanin in the noradrenergic cortical/hippocampal nerve terminals (Xu et al., 1998), that are the projections of the LC neurons: evidence for the view that release of a neuropeptide leads to increased synthesis of transcript and peptide. NPY expression in LC has, contrasting galanin, not been reported to be regulated by stress, but NPY mRNA is increased after reserpine administration (Gundlach et al., 1990a).

An involvement of LC in depression-like behavior has been studied by Weiss and colleagues focused on a link with the ascending mesencephalic dopamine system (Weiss et al., 1981, 1996, 1998, 2005). They based their experiments on the study by Grenhoff et al. (1993) showing that burst stimulation of LC inhibits DA neurons in the ventral tegmental area (VTA) (a.k.a the A10 group) (Dahlstrom and Fuxe, 1964). Weiss and colleagues have found that infusion of galanin into the VTA reduced exploratory behavior and increased immobility in the Porsolt test (a.k.a. Forced Swim Test, FST), an increase that was blocked by the galanin antagonist galantide. These findings link the LC-galanin system to studies showing involvement of the VTA and the reward system in stress and depression (Everitt and Robbins, 2005; Nestler and Carlezon, 2006; Thomas et al., 2008; Nestler, 2015; Pena et al., 2017).

The LC is involved in other mood-related behaviors such as addiction and reward (Maldonado and Koob, 1993), and galanin plays a role also in this context (Picciotto, 2008; Genders et al., 2018a). Thus, galanin binding and levels of GalR1 mRNA are increased in the LC during opiate withdrawal (Zachariou et al., 2003). Moreover, galanin-knockout mice exhibit more pronounced signs of opiate withdrawal, and galanin and the galanin ligand galnon both attenuate opiate reward and signs of withdrawal (Zachariou et al., 2003).

## The Galanin System in the Normal Human Brain

The distribution of galanin in the ‘normal’ human brain has been studied with RIA (Bennet et al., 1991; Barde et al., 2016), IHC (Chan-Palay, 1988a,b, 1990; Gentleman et al., 1989; Kowall and Beal, 1989; Beal et al., 1990; Kordower and Mufson, 1990; Kordower et al., 1992; Gabriel et al., 1994) and ISH (Miller et al., 1999; Le Maitre et al., 2013). In addition, the receptor distribution was analyzed with autoradiographic ligand binding methodology (Kohler et al., 1989; Kohler and Chan-Palay, 1990). Here, recent results obtained with ISH, qPCR and RIA on the galanin system in the LC and some other regions are summarized (Le Maitre et al., 2013; Barde et al., 2016). These studies were based on the identified gene sequences of the *human* galanin peptide and receptors (Evans and Shine, 1991; Jacoby et al., 1997; Lorimer et al., 1997; Fathi et al., 1998; Kolakowski et al., 1998; Smith et al., 1998).

### *In situ* Hybridization

**FIGURE 8:**
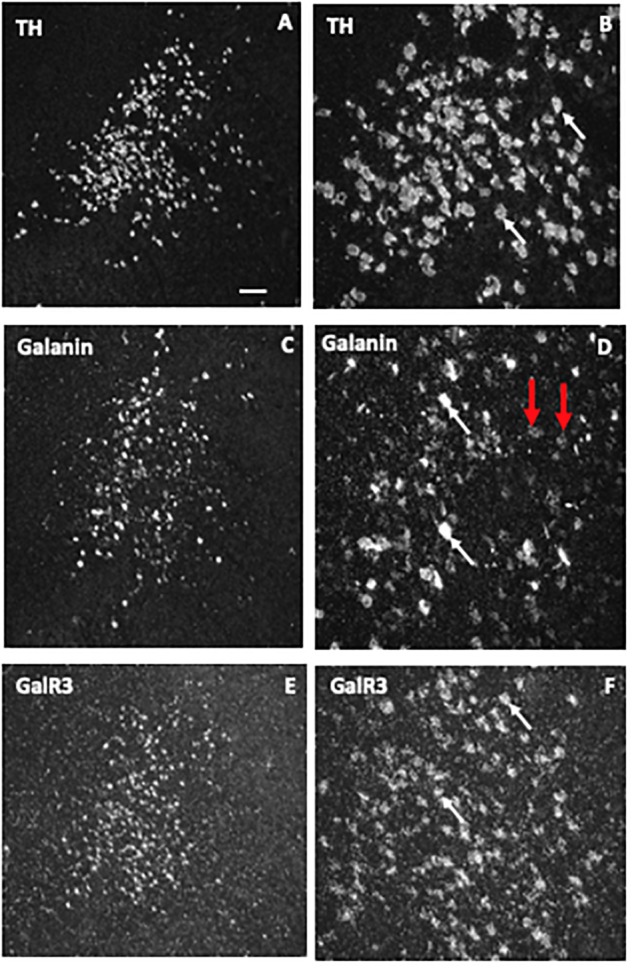
Dark-field ISH photomicrographs showing the distribution of transcripts for tyrosine hydroxylase (TH), galanin, and GalR3 in the locus coeruleus. The three markers TH **(A,B)**, galanin **(C,D)**, and GalR3 **(E,F)** show overlapping distribution patterns. TH and GalR3 transcript levels seem approximately similar in all cells. In contrast, there is variability in the strength of the signal for galanin mRNA (white arrow points to neurons with a strong signal, red ones to such with a weak signal). Exposure time: TH, 10 days; galanin, 4 weeks; GalR3, 8 weeks. This difference in exposure time transcript reflects difference in transcript levels, that is GalR3 mRNA levels are very low. Reprinted from Le Maitre et al. (2013) [Scale bars: 200 μM for **(A,C,E)**; 50 μM for **(B,D,F)**].

The ISH analysis of the human LC (Le Maitre et al., 2013) revealed expression of TH, the rate-limiting enzyme for catecholamine synthesis in presumably all noradrenergic neurons (Figures 8A,B), galanin mRNA in many LC neurons (Figures 8C,D) and GalR3 mRNA in many, perhaps all neurons (Figures 8E,F), the latter two overlapping with the TH distribution. However, whereas the levels of TH and GalR3 mRNA are relatively similar in all cells, there was a large variation in the intensity of the galanin mRNA signal (c.f. Figures 8A,B,E,F with Figures 8C,D). This likely reflects the fact that galanin is a releasable molecule and that individual neurons are in different activity states. Note that the exposure time of the emulsion dipped slides is very different for the three markers (10 days for TH versus several months for GalR3), reflecting differences in mRNA levels (Figures 8A–F). Thus, GalR3 mRNA levels are very low, in agreement with low levels in rat (Mennicken et al., 2002) and potentially undetectable levels in mouse (Lein et al., 2007). In fact, GalR3 transcripts could only be visualized in human brains with very short post mortem delays prior to freezing (2–4 h).

### RIA, qPCR and DNA Methylation

Barde and colleagues analyzed > 200 *postmortem* brain samples from ‘normal’ (and depressed, see below) female and male subjects, including the following regions: in addition to LC, dorsolateral prefrontal cortex (DLPFC), anterior cingulate cortex (ACC), DRN and the medullary raphe nuclei (MRN)^6^ (Barde et al., 2016). Three methods were applied: RIA to monitor peptide levels, qPCR for transcript levels, and pyrosequencing to analyze DNA methylation. Comparable information is presented for ligand (galanin) (Table 1A) and transcripts (Table 1B) of galanin and GalR1-3 in the *normal* (control) and (‘depressed’) male and female brain (Barde et al., 2016).

**Table 1:** **(A,B)** Concentration of galanin peptide (pmol/mg ± SEM) analyzed by RIA **(A)** and raw Ct values ± SEM monitored by qPCR **(B)** for male and female control and suicide samples from five brain regions.

	DLPFC	ACC	DRN	LC	MRN
**(A) Region**					
Male Con	3.9 ± 1.2	5.4 ± 1.2	61.6 ± 13.7	31.8 ± 7.5	15.6 ± 2.3
Male DS	2.7 ± 0.5	4.9 ± 1.0	68.1 ± 11.6	*37.4* ± *10.7*	18.7 ± 4.8
Female Con	1.7 ± 0.4	6.4 ± 1.8	80.6 ± 10.2	43.4 ± 6.3	15.5 ± 3.5
Female DS	1.3 ± 0.3	6.0 ± 1.3	81.6 ± 5.2	*67.7* ± *7.5*	13.5 ± 2.9
**(B) Region**					
Galanin	27.1 ± 0.2	27.5 ± 0.1	26.2 ± 0.4	24.6 ± 0.5	25.2 ± 0.4
GalR1	25.7 ± 0.2	28.6 ± 0.1	27.0 ± 0.4	26.4 ± 0.6	26.9 ± 0.4
GalR2	31.4 ± 0.2	31.6 ± 0.2	29.3 ± 0.2	32.3 ± 0.1	34.6 ± 0.2
GalR3	33.2 ± 0.1	33.5 ± 0.2	31.5 ± 0.4	31.4 ± 0.6	31.9 ± 0.4

*From Barde et al. (2016). Con, controls, DS, depressed suicides; DLPFC, dorsolateral prefrontal cortex; ACC, anterior cingulate cortex; DRN, dorsal raphe nucleus; LC, locus coeruleus; MRN, medullary raphe nuclei; Gal, galanin. Italics indicate significantly lower galanin levels in male vs. female depressed suicides.*

When evaluating the results it should be noted that peptide levels in normal brains mostly reflect peptide present in nerve terminals and less so in cell bodies. Animal studies have shown that galanin peptide (like many other peptides) can best be detected in cell bodies after inhibition of axonal transport by colchicine (Rokaeus et al., 1984; Skofitsch and Jacobowitz, 1985; Melander et al., 1986c). In contrast, peptide transcripts are easily seen in cell bodies/dendrites, and often confined to these neuronal compartments.

With RIA, marked regional differences in galanin levels were observed, being highest in DRN (> LC > MRN > ACC = DLPFC), whereby DRN levels were 2 times higher than in LC and ∼12 times higher than in ACC/DLPFC. The qPCR analysis revealed the highest galanin mRNA levels in LC (reflecting the many positive cell bodies), about 4-fold higher than in DRN, and 6-fold higher than DLFPC, in agreement with the ISH results (Le Maitre et al., 2013) (Table 1A). Thus, the results from the LC suggest that there is a good translation from mRNA to peptide. The RIA results are generally in agreement with IHC studies on the primate brain (Kordower et al., 1992), and on the rat brain, when analyzed with RIA (Skofitsch and Jacobowitz, 1986) and IHC (Skofitsch and Jacobowitz, 1985; Melander et al., 1986b; Merchenthaler et al., 1993).

The results are also in line with the cited immunohistochemical results in the rat with a high density of galanin-positive nerve terminals in the DRN, and fewer in the LC (Skofitsch and Jacobowitz, 1985; Melander et al., 1986b; Merchenthaler et al., 1993). It is likely that galanin in cortical areas is present in thin and rather sparse afferents to the cortex, possibly originating in LC, as is the case in rat (Xu et al., 1998) and also in local neurons (see below).

With regard to receptors only transcripts and methylation were studied. This is partly due to a lack of specific antibodies for the galanin receptors, as discussed (Lu and Bartfai, 2009; Brunner et al., 2018), and there was no attempt to use Western blotting, Elisa or IHC for receptors. The strongest signal by far was noted for GalR1 mRNA, with the highest levels seen in DLPFC (> LC > MRN = DRN > ACC) (Table 1B). GalR1 levels in DLFPC were 2 times higher than in LC, and the GalR1 mRNA levels in DLFPC were *8-fold* higher compared to the ‘adjacent’ ACC. GalR2 mRNA levels were in general considerably lower in LC (64-fold lower than GalR1). The GalR3 mRNA levels were low, although four times higher in the lower brain stem (LC, DRN) than in cortical regions, in agreement with the ISH results showing that GalR3 mRNA is present in NA neurons in LC, and possibly in 5-HT neurons in the DRN (Le Maitre et al., 2013).

In summary, GalR1 mRNA is the most prominent galanin receptor transcript in the human brain, including cortical regions. This is in agreement with early studies on human *postmortem* brain with iodinated galanin and autoradiography that revealed a distinct cortical signal (Kohler et al., 1989; Kohler and Chan-Palay, 1990), thus likely representing GalR1. The results differ from rat, since the early ligand binding studies in adult rat lacked binding in dorsal cortical areas (Skofitsch et al., 1986; Melander et al., 1988), and since GalR1 and -R2 mRNA levels are low (O’Donnell et al., 1999; Burazin et al., 2000). Thus, cortical receptor levels may represent another species difference not only between rat and human, but also between rat and mouse (Jungnickel and Gundlach, 2005). However, overall, GalR1 is also the most prominent galanin receptor in the rat brain (O’Donnell et al., 1999; Burazin et al., 2000) and likely also in mouse brain (Hohmann et al., 2003; Jungnickel and Gundlach, 2005; Lein et al., 2007). Waters and Krause (2000) monitored the levels of transcript for all three galanin receptors in the rat brain: GalR1 is highest in amygdala and spinal cord, whereas in cortex GalR2 > GalR1 > GalR3, and in hippocampus GalR2 > GalR1 = GalR3. In that study values were expressed as mean pg/25 μg total RNA.

The interpretation of the human qPCR results is not straight forward. For example, there is a lack of knowledge of the cellular localization of the transcripts in the prefrontal cortex regions, i.e., these areas have not been studied with ISH. It is likely that the transcripts are present in neurons, but a glial localization cannot be excluded (Butzkueven and Gundlach, 2010). Under certain circumstances galanin is expressed in specialized glial cells, e.g., after colchicine treatment alone or after spreading depression (Xu et al., 1992; Shen et al., 2003, 2005). The colchicine-induced signal was abolished by thyroidectomy (Calza et al., 1998). However, the results strongly suggest that galanin, and possibly all three galanin receptors, or at least GalR1, are expressed locally in cells, likely in cortical neurons. In contrast, ISH results *are* available for DRN and LC and reveal neuronal localization (Le Maitre et al., 2013). In particular, the results on the LC offer a possibility to form a hypothesis about the galanin system in this nucleus being involved in stress and genesis of depression, as discussed below.

Overall major differences exist between species, both with regard to galanin and galanin receptor expression, whereby galanin signaling seems to be more important for dorsal cortical functions in the human brain than in rodents. In contrast, in the rat ventral cortical areas, like entorhinal and piriform cortices, have abundant galanin receptor expression (Skofitsch et al., 1986; Melander et al., 1988; O’Donnell et al., 1999; Burazin et al., 2000), suggesting involvement in limbic processes.

## Depressive Disorders

Major depressive disorder (MDD) is a common and serious disease afflicting up to 2–5% (12-month prevalence; lifetime prevalence 10–15%) of the population worldwide, and women being more susceptible than men. Thus, MDD is a leading cause of disability worldwide associated with much suffering and major costs for society (Murray and Lopez, 1997; Kessler et al., 2003; Wittchen et al., 2011; Ferrari et al., 2013; World Health Organization [WHO], 2013). Adverse life events usually precede depression episodes, and experiences of physical and emotional abuse during early childhood and parental neglect are important predisposing vulnerability factors, strongly indicating that environmental psychosocial stressors are essential in pathogenesis (Kendler, 2012, 2013; Lutz et al., 2017; Tanti et al., 2017). The heritability is significant, about 35% (Sullivan et al., 2000). Interaction of genetic and environmental factors including stressful life events plays a major role in the development of MDD (Nestler et al., 2002; Akil, 2005; de Kloet et al., 2005; McEwen, 2007; McEwen et al., 2015). Epigenetic mechanisms through altered DNA methylation (Meaney and Ferguson-Smith, 2010; Zhang and Meaney, 2010) are probably involved, leading to stable changes in brain function that may underlie the psychopathology (Labonte et al., 2013; Vialou et al., 2013).

Over the last several decades two major hypotheses of the cause of unipolar depression have dominated, clinically associated with catecholamines (Bunney and Davis, 1965; Schildkraut, 1965; Schatzberg and Schildkraut, 1995) and with serotonin (Coppen, 1968; Maes and Meltzer, 1995). Pharmacological management of depression therefore often involves drugs that target the monoamine transporters, which include SSRIs, the transporter for noradrenaline (NA) (NRIs) or a combination of both (SNRIs) (Gardier et al., 1996; Mongeau et al., 1997; Millan, 2006), as well as a number of other medications (Berton and Nestler, 2006; Millan et al., 2015b). However, the therapeutic efficacy of these antidepressants is hampered by a slow onset of action, a limited response rate and considerable side effects (Montgomery, 2006; Trivedi et al., 2006). Of particular importance is the treatment resistant depression which affects some 20% of afflicted subjects (Akil et al., 2018). These issues have led to an intensive search for novel therapeutic approaches for MDD (Berton and Nestler, 2006; Artigas, 2015; Akil et al., 2018) (and see below), including targeting receptors for neuropeptides (Maubach et al., 1999; Hokfelt et al., 2003; Holmes et al., 2003; Nemeroff and Vale, 2005; Griebel and Holsboer, 2012), the most diverse family of brain messenger molecules (Burbach, 2010).

## The Galanin System and Depression in Humans

The evidence from animal experiments described led us to explore to what extent galanin may be involved in MDD and other mood disorders, and whether results from the analysis of human brain can guide the search for new antidepressants.

### Genetic Variations in the Galanin System in Depression

**FIGURE 9:**
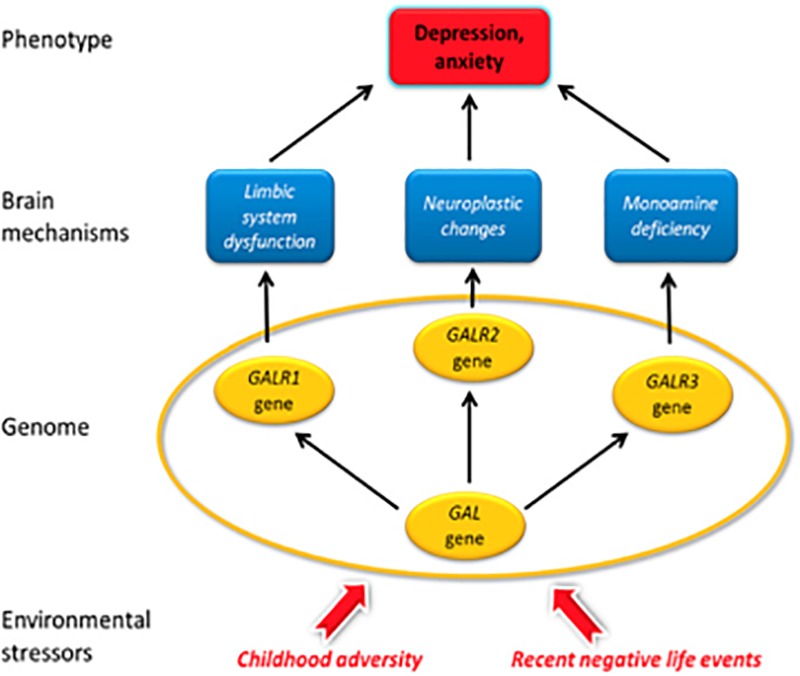
Galanin mechanisms hypothetically involved in MDD in humans. Galanin and its receptors are colocalized in some monoaminergic neurons in the brain. The galanin system is highly sensitive to experimental and naturalistic stressors. Stress-induced activation of the galanin system represents the first phase in the development of depression. Recent analysis of human brain has shown that the Gi protein-coupled GalR3 (and not GalR1 as in rodents) is the main galanin receptor in noradrenergic neurons in the locus coeruleus and probably the dorsal raphe nucleus and that the Gi protein-coupled GalR1 is the main receptor in the forebrain. Antidepressive effects may be achieved by (i) GalR3 antagonists, by reinstating normal monoamine turnover in LC neurons in the lower brainstem projecting to the forebrain, and by (ii) GalR1 antagonists in the forebrain by normalization of limbic system activity, or by (iii) agonists at GalR2, a Gq protein-coupled receptor, promoting neuroprotection. A candidate gene analysis suggests that GalR1 risk variants may compromise galanin signaling during childhood, whereas GalR2 signaling may be influenced by recent negative life events. In addition, all four galanin system genes have relevant roles in the development of depression-related phenotypes in those persons who were highly exposed to life stressors. From Juhasz et al. (2014).

A candidate gene study of a cohort of European White ethnic origin totaling 2,361 from Manchester, United Kingdom and Budapest, Hungary was carried out (Juhasz et al., 2014) and revealed that variants in genes for galanin and its three receptors confer increased risk of depression and anxiety in people who experienced childhood adversity and/or recent negative life events (Figure 9). Genetic factors were only relevant in the moderate or high stress exposure groups when applying Bayesian multivariate analysis (Juhasz et al., 2014; Gonda et al., 2018). The rank order of the relevance of gene polymorphisms was GalR2 > GalR3 > GalR1 > galanin, with strong relevance for the first three in the moderately or highly exposed persons by recent negative life events in the last 12 months. All four were more relevant than the serotonin transporter gene-linked polymorphic region (5-HTTLPR) of the serotonin transporter gene. The effects were seen in the Manchester and the Budapest population, and in both males and females. This impact was seen only if taking stress into account, after medium and strong stress (the GalR2 gene) or strong stress (the GalR3, GalR2 and galanin genes), underlining the importance of environmental factors. In addition, the GalR2 gene polymorphism was more relevant than candidate gene polymorphisms of the genes for BDNF, the serotonin 1A receptor (HTR1A), the cannabinoid 1 receptor (CB1) and the serotonin 2A receptor (HTR2A) in the moderately stress exposed subjects (Gonda et al., 2018). The traditional analysis based on general linear models confirmed the gene-environment interaction; namely, no main effect of genes, but a significant modulatory effect of environment-induced development of depression were found.

Evidence for collaboration between small neurotransmitter and neuropeptide in the development of depression was also identified in this study, namely a gene–gene–environment interaction between the GalR2 and 5-HTTLPR genes in strongly exposed persons (Gonda et al., 2018). This could be of interest regarding antidepressant drug targets. The expression of the GALR2 polymorphism is about 2.5 times higher compared to 5-HTTLPR. Currently, the most frequently used antidepressants are the SSRIs, NRIs and SNRIs. Preliminary pre-clinical results suggest that an SNRI (venlafaxine) does not alter the transcript levels of galanin and its receptors (Petschner et al., 2016). Other studies show that chronic treatment with SSRIs increases galanin mRNA levels in various brain regions (Christiansen et al., 2011; Rovin et al., 2012; Yamada et al., 2013). The higher relevance of the GalR2, GalR3 and GalR1 gene polymorphisms in stress-induced depression and the galanin system-independent effects of the currently used antidepressants suggest that novel antidepressants acting on GalR1-3 could be developed. Such compounds could perhaps be more effective in SSRI/SNRI non-responders.

### Galanin Versus 5-HT Transporter in Depression

For a long time, it has been assumed that there is an interaction between stressful life events and a polymorphism in the promoter region of the 5-HT transporter (5-HTT) gene (SLCA4) (Lesch et al., 1996; Caspi et al., 2003). In the Newmood cohort this effect was weak and not significant in most comparisons, when corrections for multiple testing were applied (Juhasz et al., 2015). Furthermore, Bayesian relevance analyses consistently failed to show relevance for 5-HTTLPR (Juhasz et al., 2014; Gonda et al., 2018). Parallel to these findings, a recent large meta-analysis could not confirm an interaction (Culverhouse et al., 2018). The findings on the galanin system provide evidence for a more robust and relevant effect of galanin system genes compared to 5-HTTLPR of the serotonin transporter gene (Juhasz et al., 2014; Gonda et al., 2018). The 5-HTT and GalR2 receptor act jointly in the development of depression (Gonda et al., 2018).

### Other Genetic Studies

Involvement of galanin in depression is further supported by a gender-specific association of galanin polymorphisms with antidepressant treatment response (Unschuld et al., 2010) and by a study reporting an association of galanin and MDD in the Chinese Han population (Wang et al., 2013). In addition, the first large genome-wide association study (GWAS) obtained a suggestive association of *GAL* with MDD using a gene based test, which retained low association p-values in two additional independent cohorts (Wray et al., 2012). A very large GWAS failed to identify risk genes (Major Depressive Disorder Working Group of the Psychiatric Gwas Consortium, 2013), but recent advances in large MDD GWAS studies resulted in several SNPs being associated with MDD (e.g., Hyde et al., 2016; Okbay et al., 2016; Xiao et al., 2017; Wray et al., 2018), and provided further evidence that genetic risk for depression is a continuous measure that translate environmental adversities into depressive symptoms. Taken together, the genetic analysis of the four members of the galanin system genes are complemented by a study on *postmortem* brains from depressed suicides (Barde et al., 2016), strengthening an involvement of galaninergic mechanisms in depression, as discussed below.

### Multiple Changes in the Galanin System in MDD

**FIGURE 10:**
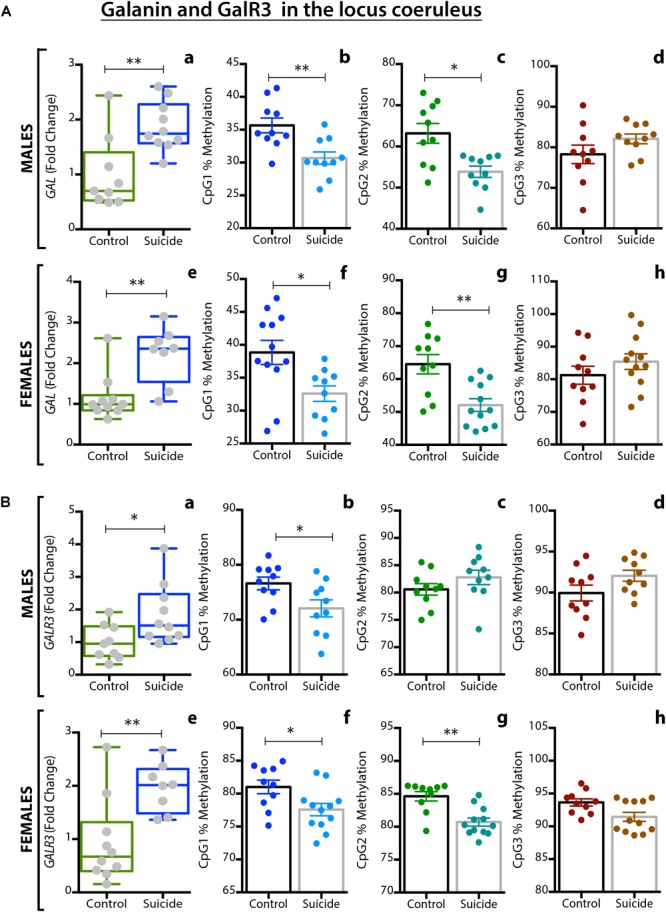
**(A,B)** Alterations in galanin **(A)** and GalR3 **(B)** gene expression and DNA methylation in the locus coeruleus (LC) of male and female depressed subjects who committed suicide, as compared to matched controls. **(a,e)** Expression levels of the two genes in the LC of male **(a)** and female **(e)** controls and depressed suicide (DS) subjects. **(b–d,f–h)** Percentage of DNA methylation levels at individual CpG sites of the two genes in male **(b–d)** and female **(f–h)** controls and DS subjects. All data are presented as mean ± SEM; males: *n* = 10 controls, 10 DS subjects; females: *n* = 12 controls, 10 DS subjects. Significant differences between DS subjects and controls are indicated: ^∗^*P* < 0.05, ^∗∗^*P* < 0.01. CON, controls. From Barde et al. (2016).

*Differences* in levels of galanin peptide, and of transcripts for, and DNA methylation of, galanin and GalR1-3 between MDD patients and matched controls were observed in an analysis of > 200 *postmortem* samples from five male and female brain regions (DLPFC, ACC, DRN, LC, and MRN). The significant and selective differences and changes in the galanin system in depressed versus control brains are summarized in Table 2. The most pronounced changes were observed for galanin and GalR3 in the DLPFC, and for galanin and GalR3 in the DRN and LC, in males and females (examples of results in Figures 10A,B). In DRN and LC there was an *upregulation* of the transcripts, paralleled by a *decrease* in DNA methylation. The decrease in methylation in galanin and GalR3 was most pronounced in female DRN and in male and female LC. In DLPFC, galanin mRNA levels were *decreased* in males and *increased* in females, the only distinct sex difference observed in the study. The changes in GalR1 were also increased and confined to three regions, DLPFC (male and female), DRN (male) and MRN (male), versus no change in LC. No differences were seen with regard to GalR2, except a decrease in MRN, included as a control region. The complete lack of changes in ACC contrasts the dramatic alterations in DLPFC, both regions belonging to the prefrontal cortex complex.

**Table 2:** Overview of mRNA and DNA methylation changes.

		Galanin				GalRl					GalR2					GalR3			
		IDNA methylation				gDNA methylation					gDNA methylation					gDNA methylation			
Regions	Sex	mRNA	CpGl	CpG2	CpG3	mRNA	CpGl	CpG2	CpG3	CpG4	mRNA	CpGl	CpG2	CpG3	CpG4	mRNA	CpGl	CpG2	CpG3
DLPFC	Males	↓↓	↑		↑↑	↑	–	–	–	–	–	–	–	–	–	↓↓	–	–	–
	Females	↑	↑	↓	–	↑	–	–	–	–	–	–	–	–	–	–	–	–	–
ACC	Males	–	–	–	–	–	–	–	–	–	–	–	–	–	–	–	–	–	–
	Females	–	–	–	–	–	–	–	–	–	–	–	–	–	–	–	–	–	–
DRN	Males	↑↑	–	↓	–	↑	–	–	–	–	–	–	–	–	–	↑	–	–	–
	Females	↑↑	–	–	–	–	–	–	–	–	–	–	–	–	–	↑↑	↓↓	↓↓	–
LC	Males	↑↑	↓↓	↓	–	–	–	–	–	–	–	–	–	–	–	↑	↓	–	–
	Females	↑↑	↓	↓↓	–	–	–	–	–	–	–	–	–	–	–	↑↑	↓	↓↓	–
MRN	Males	↑	–	–	–	↑	–	–	–	–	↓↓	–	–	–	–	↑	–	–	–
	Females	↑	–	–	–	–	–	–	–	–	–	–	–	–	–	↑	–	–	–

*From Barde et al. (2016). The arrows represent statistical significance, where upward arrow signifies increase in gene expression and methylation status and vice-versa. One arrow signifies *P* < 0.05 and two arrows stand for *P* < 0.01. DLPFC, dorsolateral prefrontal cortex; ACC, anterior cingulate cortex; DRN, dorsal raphe nucleus; LC, locus coeruleus; MRN, medullary raphe nuclei.*

With regard to methylation, changes were always opposite to those in transcript levels. This is in agreement with the general view that methylation suppresses transcript synthesis (Moore et al., 2013). The results lend further support for an involvement of epigenetic mechanisms in MDD (Mill and Petronis, 2007; Machado-Vieira et al., 2011; Vialou et al., 2013; Lolak et al., 2014; Lopizzo et al., 2015; Saavedra et al., 2016; Hoffmann et al., 2017; Nagy et al., 2018).

Taken together, the results suggest that galaninergic mechanisms, in several brain regions, are involved in MDD, and that epigenetic changes mediated by DNA methylation play an important role, in agreement with a candidate gene study (Juhasz et al., 2014).

## Involvement of Galanin in Depression and Resilience – a Hypothesis

The LC in humans is a compact (but less so than in rodents), ‘blue’ (pigmented) nucleus consisting of a total (both sides) of around 50,000 neurons (German et al., 1988; Baker et al., 1989; Chan-Palay and Asan, 1989; Miller et al., 1999; Szot et al., 2000). As in other mammalian species studied, galanin is expressed in a large proportion of the human noradrenergic neurons (Chan-Palay et al., 1990; Kordower et al., 1992; Miller et al., 1999; Le Maitre et al., 2013), suggesting conservation during evolution. However, there are differences with regard to receptors. Thus the GalR3 receptor seems to be the most prominent receptor in the LC, contrasting the robust expression of GalR1 and GalR2 in the rat LC (O’Donnell et al., 1999; Burazin et al., 2000). Neither GalR1 nor -R2 mRNA was, surprisingly, detected with ISH in human NA neurons, although a GalR1 signal was seen in the LC region and in other regions, suggesting that the probe was functional. However, it cannot be excluded the NA LC neurons contain lower levels of GalR1 than other types of neurons and thus escaped detection. Thus, a distinct species difference seems to exist. Another apparent species difference was the possible lack of galanin expression in the human 5-HT neurons (Le Maitre et al., 2013), as is the case also in the mouse (Larm et al., 2003; Kuteeva et al., 2004; Lein et al., 2007; Fu et al., 2010), versus a robust expression of galanin in rat 5-HT neurons (Melander et al., 1986c; Fuxe et al., 1990; Priestley et al., 1993; Xu and Hokfelt, 1997).

**FIGURE 11:**
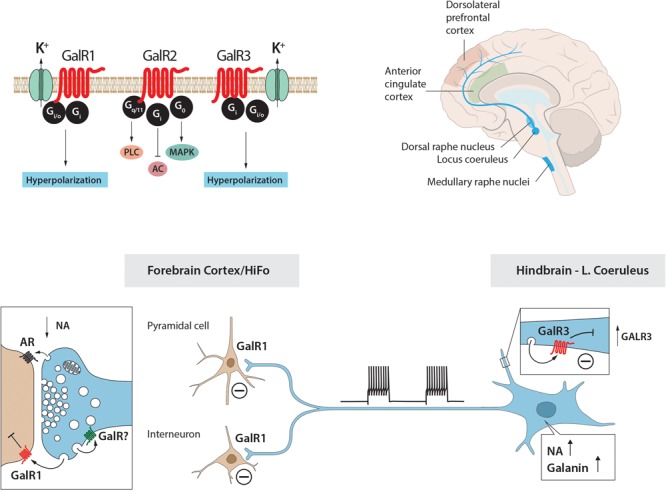
The galanin–locus coeruleus (LC) system in stress and depression: A hypothesis. The hypothesis is built on animal (rat) experiments showing that (i) galanin and GalR1 **(top left)** are present in LC NA neurons; (ii) galanin mRNA levels are increased during stress; (iii) galanin via GalR1 autoreceptors inhibits firing of LC neurons; and (iv) indirect evidence that galanin can be released from soma-dendrites of LC neurons. The second corner stone is results from two studies on human *postmortem* brain with ISH and qPCR. Five regions from *postmortem* brains from depressed subjects who committed suicide and controls were studied and are shown, including LC that projects to anterior cingulate and dorsolateral prefrontal cortices **(top right)**. The results show that also in humans (i) the NA LC neurons express in any case galanin and GalR3 **(top left)**. GalR1 and GalR3 probably have similar transduction mechanisms **(top left)**. Under ‘normal’ firing only noradrenaline is released in forebrain. A situation after severe stress is depicted in the **lower panel**: LC neurons burst fire (lower panel, **middle**), NA and galanin are released from nerve endings in cortex (**lower panel**, left) and dendrites in the LC (**lower panel**, right), the latter in an attempt to prevent overexcitation (a resilience mechanism). To replace released peptide, galanin transcript levels and synthesis increase, and also GalR3 is upregulated (**lower panel**, right). The increased release, together with elevated galanin and GalR3 levels, result in a too strong inhibition and decreased NA levels in the forebrain (maladaptation) (**lower panel**, left), possibly contributing to depressive symptoms. HiFo, hippocampal formation. Drawing by Mattias Karlén.

How and when neuropeptides and classic transmitters are released has been explored (see Lundberg and Hokfelt, 1983; Hokfelt, 1991; Lundberg, 1996). Neuropeptides, stored in LDCVs, are mainly released when neurons are firing at a high rate or in bursts, e.g., during stress. This release occurs extrasynaptically, and not only from nerve endings but also from soma/dendrites. Merging this information and the results from MDD patients (Barde et al., 2016) it is possible to generate a hypothesis how depression in humans, likely a (stress-related) subtype of MDD, may develop (Figure 11): Under normal circumstances LC neurons fire at low frequencies, releasing NA in cortical regions, acting on post- and pre-synaptic adrenoceptors. Under stress, when LC neurons fire in bursts, also galanin will be released, together with NA, from nerve endings and soma/dendrites of the NA LC neurons, galanin acting on somatic/dendritic GalR3 autoreceptors.

GalR3 is, like GalR1, inhibitory (Smith et al., 1998) and causes hyperpolarization of the LC neurons, the purpose being to act as a ‘brake’ to prevent overexcitation, to keep the system in balance. This is similar to the proposed function of the 5-HT1A receptor as a “safety valve” of 5-HT neurons (Celada et al., 2013). As a consequence of increased firing and increased galanin release, synthesis of new peptide is initiated, reflected in increased mRNA levels. The fact that in dendrites sites of synthesis and release are close allows for rapid replacement. Thus, if mRNA is translated, increased galanin levels will be available for release from soma and dendrites, a feed-forward process. The additional increase in GalR3 transcript, presumably resulting in increased levels of receptor protein, could represent a robust increase in local galanin signaling. This seems unexpected, because intuitively one would expect downregulation of the receptor, following elevated levels of ligand. However, the inhibition may be strong and long-lasting leading to depletion of NA in the forebrain. The results suggest that mood disorders may be a consequence of a maladaption, an allostatic load (McEwen, 2003).

It may be emphasized that the prefrontal cortex has not been included in the discussion, even if significant changes in levels of galanin transcripts and methylation were recorded in this region of depressed subjects (Barde et al., 2016). This is because lack of knowledge about the cellular localization of the galanin system in this brain area. Finally, a similar scenario for an anti-depressive role of a GalR3 antagonist could be sketched for the 5-HT neurons in the DRN, since the galanin and GalR3 transcripts are upregulated both in the male and female DRN region from depressed patients who committed suicide, paralleled by decreased DNA methylation of the GalR3 gene in the female depressed subjects (Barde et al., 2016).

Of note, in the Barde et al. (2016) study the end stage of a mostly long development of the disorder is recorded, where all ‘resources’ have been mobilized to prevent overexcitation: increased ligand release plus increased receptor availability. Alternatively, the situation may reflect changes beyond patho-physiological regulatory mechanisms, especially when considering the considerable time it takes for depression to arise^7^.

### Resilience

Even if many humans are exposed to stress of various types and intensity, only comparatively few develop depression, thus displaying resilience to stress (Nestler et al., 2002; Southwick et al., 2005; Han and Nestler, 2017). Resilience appears to represent an active process involving several systems, including not only the mesolimbic dopamine neurons (Han and Nestler, 2017) but also other systems, such as the noradrenergic LC neurons (Charney, 2004; Feder et al., 2009; Krystal and Neumeister, 2009; Sciolino et al., 2015; Valentino and Van Bockstaele, 2015; Isingrini et al., 2016). Specific molecules, e.g., BDNF and neuropeptides like opioids and CRF, have also been implicated (Russo et al., 2012). Of particular interest in the present context is NPY (Kask et al., 2002; Morgan et al., 2002; Heilig, 2004; Krishnan et al., 2007; Zhou et al., 2008; Domschke et al., 2010; Cohen et al., 2012; Sabban et al., 2016; Kautz et al., 2017), a neuropeptide discovered in the Mutt laboratory (Tatemoto et al., 1982). NPY may be involved in the control of LC signaling in a similar way as galanin, but this will not be discussed further here, because limited information is available on the expression of NPY and NPY receptors in the brain of normal subjects and subjects who committed suicide.

The present data suggest that the GalR3-mediated ‘brake’ on the LC neurons is part of the resilience ‘machinery’ in humans. This is in agreement with animal experiments on rats by Sciolino et al. (2015) who show that exercise increases galanin levels in LC, and that exposure to stress reduces open arm exploration in sedentary rats. But this effect is not seen in exercise rats – and not in rats treated chronically with galanin given intraventricularly (i.c.v.); and it could be blocked by chronic administration of the galanin antagonist M40. Thus, increased galanin levels, presumably in LC, promotes resilience. This is also suggested by earlier animal experiments, as summarized by Sciolino et al. (2015): i.c.v. galanin protects against anxiety under stressful conditions (Bing et al., 1993), but not in the absence of stress (e.g. Holmes et al., 2005); i.c.v. M40 blocks fluoxetine-induced activity in the FST (Lu et al., 2005a); and transgenic mice overexpressing galanin under the dopamine B-hydroxylase promoter (the GalOE/D mouse), i.a. in LC, are protected against stress (Holmes et al., 2002).

The resilience hypothesis is further supported by comparison of the GalOE/D mouse (Steiner et al., 2001) with a mouse overexpressing galanin under the platelet growth factor B (PDGFB) promoter (the GalOE/P mouse) (Holmberg et al., 2005). Analyzing the effect of swim stress with microdialysis, it was shown that NA release in the forebrain is much lower in the GalOE/D than in the GalOE/P mouse (Yoshitake et al., 2004). The histochemical/qPCR analyses revealed that the galanin mRNA levels in the LC are 5 times higher in the GalOE/D than in wild type mice (Steiner et al., 2001). On the other hand, the GalOE/P mouse has a lower galanin expression in the LC and in (noradrenergic) nerve terminals in the forebrain (Kuteeva et al., 2004; Yoshitake et al., 2004). One possible explanation is that the higher galanin levels in the GalOE/D mouse suppress NA release in the forebrain by autoinhibition of the LC neurons, in agreement with the hypothesis.

The behavioral analyses of these two mouse lines reveal that the GalOE/P mouse displays an increased time of immobility in the FST, that is a depression-like behavior (Kuteeva et al., 2005). In contrast, there are no differences in immobility time on tail suspension between GalOE/D and wild type mice (Holmes et al., 2005). One interpretation is that the GalOE/D mouse is resilient to stress thanks to increased inhibitory galanin signaling in the LC, again, in agreement with the hypothesis.

## Drug Treatment Via Neuropeptide Receptors

The fact that neuropeptides routinely signal via GPCRs is promising from the perspective of drug development, since >30% of all prescription drugs act via such receptors (Luttrell et al., 2015; Hauser et al., 2017; Santos et al., 2017). Thus, >200 neuropeptide receptors are potential drug targets.

### Principles for Peptidergic Co-signaling

Peptide signaling in the rodent, and possibly primate, brain likely always means co-transmission with one or more small molecule transmitters – and other peptides. How could this affect treatment of patients? Are there any problems, or even advantages? Here some thoughts.

To fully block signaling at least two antagonists may be required. For example, a substance P antagonist is potentially a pain killer, since this excitatory peptide is released from nociceptors. However, the clinical tests failed for the reasons discussed (Hill, 2000; Herbert and Holzer, 2002). An obvious explanation would be that at least two additional excitatory transmitters are co-released with substance P from the same nerve endings in the dorsal horn: glutamate and CGRP, which are co-stored together with substance P in the same LDCVs (Merighi, 2002) (Figure 1E). So, blockade of one (the NK1) receptor may not be sufficient to achieve analgesia.

There could be advantages with peptide transmitters: First, neuropeptides are ’weak’ messengers. Thus, an antagonist will not have the potentially detrimental effects of blocking ‘strong’ and functionally essential fast transmitters, like those for GABA and glutamate. Although glutamate antagonists have many obvious indications for treating disease, it has been difficult to develop clinically efficacious and safe medicines. For example, glutamate is the major transmitter in nociceptors, and glutamate ligands have been strong candidates for pain treatment (Neugebauer, 2007), but glutamate antagonists like ketamine have yet to emerge as a clinically safe and widely used treatment alternative for pain (Bell et al., 2017). This said, and important in the context of the present review (MDD), the introduction of ketamine and ketamine analogs causing rapid antidepressant effects in subjects with treatment resistant depression has been, to say the least, an exciting advance (Zarate et al., 2006; Abdallah et al., 2015; Lener et al., 2017).

Furthermore, if neuropeptides are only released when neurons are firing at high frequency or firing in bursts, then antagonists would only have an effect under these circumstances, that is only affecting an activated system. For example, galanin is present in >20 nuclei in the rat brain. However, stress may primarily activate LC and 5-HT neurons, which in rats may be the only neurons releasing galanin. And an antagonist will consequently antagonize only the effects of galanin released from these two systems. The remaining galanin systems are silent and will therefore not be affected by the antagonist – so likely only few side effects. In contrast, a NA reuptake inhibitor will affect *all* NA neurons, resulting in increased extracellular amine levels in virtually all brain regions, probably leading to side effects. The same is of course true for SSRIs and serotonin, as well as for SNRIs and serotonin plus NA.

### Treatments via Peptidergic Mechanisms Are Effective

The discovery by the pharmaceutical company Merck of small non-peptide molecules passing the blood-brain-barrier (Uslaner et al., 2013) and acting as antagonists at orexin/hypocretin receptors (de Lecea et al., 1998; Sakurai et al., 1998) has resulted in a new medicine: Suvorex/Belsomra, approved by the federal drug administration (US FDA) for treatment of insomnia (Coleman et al., 2012; Yang, 2014): in less than 20 years from bench to patient! The small molecule co-transmitter in these orexin/hypocretin neurons is glutamate (Rosin et al., 2003), and these neurons also express the opioid peptide dynorphin (Chou et al., 2001). Furthermore, monoclonal antibodies^8^ to calcitonin gene-related peptide (CGRP) (Aimovig, erenumab), a peptide also present in nociceptors (Rosenfeld et al., 1983), are now approved by FDA and EMA for treatment of migraine (Silberstein et al., 2017; Edvinsson et al., 2018). In fact, antibodies to the CGRP *receptor* and CGRP *antagonists* are also efficacious in treatment of migraine (Silberstein et al., 2017; Edvinsson et al., 2018). Here some 35 years passed from the discovery to the clinic. Moreover, the NK1 antagonist (Aprepitant) mentioned in relation to depression is now used for treatment of chemotherapy-induced emesis (Pendergrass et al., 2004), a serendipitous finding

### Drugs Acting on Galaninergic Signaling

It has been difficult to generate small molecules that pass the blood-brain-barrier and act on central galanin receptors. Bartfai and associates made significant contributions, starting with chimeric peptide ligands (Bartfai et al., 1992). For several years, these were important tools in the galanin field, although they did not penetrate into the brain/spinal cord from the periphery. GalR3 antagonists (SNAP 39899 and related compounds) were then the first molecule acting on the brain after peripheral administration (Swanson et al., 2005; Barr et al., 2006; Konkel et al., 2006a,b). An allosteric modulator, a GalR2 agonist passing the blood-brain-barrier, was also reported (Lu et al., 2010), followed by further GalR2 ligands (Saar et al., 2013a,b). Several overviews of the field have been published (Mitsukawa et al., 2008; Hoyer and Bartfai, 2012; Webling et al., 2012; Freimann et al., 2015).

Based on the discussion above it appears that a GalR3 antagonist is a promising candidate for treatment of depression. Experiments in rats, suggest that the GalR1 receptor in LC also is a target for treatment of addiction (Picciotto, 2008; Genders et al., 2018a). However, in humans the correct receptor may be GalR3. In fact, in two genetic studies on alcoholism, both the galanin gene (Belfer et al., 2006) and, interestingly, the GalR3 gene, but not the other two receptor genes, have been implicated (Belfer et al., 2007). Of note, a GalR3 knockout mouse exhibits an alcohol-preferring phenotype (Genders et al., 2018b).

Why would a GalR3 antagonist be an advantageous choice over reuptake blockers? Analysis of regions of the *postmortem* MDD brains and controls (Table 1) reveals upregulation of galanin and GalR3 not only in the LC but also in the DRN (Barde et al., 2016). These changes are likely associated with higher levels of released galanin and of available receptors. Thus, a GalR3 antagonist could disinhibit blockade of *two* monoamine systems critical in mood disorders and restore both NA and 5-HT forebrain levels, relieving depressed mood. Since no changes are seen in the ACC, and since galanin and GalR3 are *down*regulated in the male DLPFC, these systems are likely ‘silent’. Thus, treatment with a GalR3 antagonist may overall have a high degree of selectivity with less side effects.

## Limitations and Future Perspectives

The key message of the present review is that the neuropeptide galanin and its subtype 3 (GalR3), both coexisting in noradrenergic LC neurons, are involved in MDD as part of the resilience machinery and GalR3 as a target for treatment. The hypothesis is based on solid and reproduced animal experiments from several laboratories. However, the translation to humans represents a major ‘jump,’ only involving one single (large) experiment on postmortem brains and a supporting candidate gene study. Thus, the hypothesis needs confirmation, preferably by other laboratories and methods. GWAS reports are negative, and no support based on imaging is published. A key experiment would be to label a GalR3 antagonist and carry out both *in vitro* autoradiography and positron emission tomography to analyze GalR3 binding sites/receptors. Moreover, the results are mainly based on transcript analysis, and it will be necessary to show translation into receptor protein, both in the rodent and human brain. The final proof would be to test a GalR3 antagonist in the clinical setting, but that would require generation of new, non-toxic molecules. Therefore, the retraction of major pharmaceutical companies from the neuroscience field represents a major disappointment. Further aspects on ‘limitations’ can be found in Barde et al. (2016).

## Concluding Remarks

The discovery of new drugs for treatment of mental illness has often been the result of serendipity (Celada et al., 2013; Millan et al., 2015a). The present review suggests that results from experimental animal studies can generate hypotheses that can be further validated by examining *postmortem* brains from relevant patient groups, perhaps leading to new pharmacological treatment strategies.

In animal studies the neuropeptide galanin has shown consistent changes in expression in response to a variety of stimuli, including stress. In agreement, results on the four galanin system genes (Juhasz et al., 2014), together with a recent study conducted with *postmortem* brains from depressed suicides (Barde et al., 2016), suggest involvement of galaninergic mechanisms in depression. On the basis of these studies, it is hypothesized that galanin may, via inhibitory GalR3 autoreceptors, act as a ‘brake’ to prevent overexcitation of LC neurons, representing a *resilience* mechanism to protect against depression. Galanin is, however, only *one* factor in a comprehensive network of built-in safeguards against overexcitation of LC neurons, reflecting the functional importance of a strict control of noradrenergic LC neurons which project to virtually all parts of the central nervous system.

It is now some 45 years since fluoxetine was generated, and 35 years since the first monoamine (serotonin)-reuptake inhibitor (Zimelidine) was launched (Spector et al., 2018). Since then SSRIs, NRIs and SNRIs have been the most widely used drugs for treatment of depression. Here we speculate that an antagonist at GalR3 receptors in noradrenergic LC neurons could lead to enhanced NA release in the forebrain – and recovery from disease. Since GalR3 is also likely present and upregulated in 5-HT neurons, it is possible that the same GalR3 antagonist also could normalize 5-HT release in the forebrain as well. Such a GalR3 antagonist has been developed (Swanson et al., 2005). However, the GalR3 antagonist SNAP 37889 has shown *in vitro* toxicity (Koller et al., 2016), and clinical trials have been terminated due to safety concerns.

If a GalR3 antagonist without toxic side effects will be developed and if ever tested in the clinic, the question might arise: Why would this drug be an advantage over a combined reuptake inhibitor, like Venlafaxine? Since the GalR3 antagonist works by a different mechanism it may, hypothetically, avoid some of the well-known side effects of reuptake inhibitors by a restricted site of action, versus the reuptake inhibitors increasing monoamine levels at *all* sites in the brain. It may be further speculated that the well-known delay of onset may be avoided, since the postulated disinhibition of the NA and 5-HT neurons via GalR3 antagonism should be a fast effect, perhaps without the complex ‘compensatory’ changes occurring after treatment with SSRIs and related to the 5-HT1A receptors (Celada et al., 2013). A third consideration is the consistently higher relevance of GalR3 gene variants compared to those of the serotonin transporter in stress-related depression, which might serve as a basis of personalized treatment. To what extent treatment resistant subjects will be helped is another issue that needs to be addressed. Finally, the use of agents acting at multiple sites, e.g., blocking monoamine re-uptake plus the NK1 receptor, may represent a way forward (Millan, 2009). In this respect, perhaps a drug combining blockade of a galanin receptor with another receptor/mechanism could represent an interesting alternative?

## Author Contributions

TH and SB wrote and revised the manuscript. GB, GJ, and NM wrote key sections of the manuscript. All authors contributed to manuscript revision, and read and approved the submitted versions.

## Conflict of Interest Statement

TH has shares in Bioarctic, Stockholm, Sweden and Novo Nordisk, Copenhagen, Denmark. The remaining authors declare that the research was conducted in the absence of any commercial or financial relationships that could be construed as a potential conflict of interest.

## Footnotes

1 There are many examples: interneurons in neocortex are partly defined by (five) neuropeptides (Somogyi and Klausberger, 2005). For example, somatostatin-positive cortical interneurons are associated with gamma-rhythms (Veit et al., 2017), with the development of neuropathic pain (Cichon et al., 2017) and possibly with mental illness (Hamm and Yuste, 2016); and galanin-immunoreactive neurons in the medial preoptic area govern parental behavior (Wu et al., 2014), and in the ventrolateral preoptic nucleus they are sleep active (Gaus et al., 2002). However, in none of these studies is a functional role assigned to the peptide. Neuropeptides as phenotype marker are thus similar to calcium-binding proteins (such as parvalbumin) (Baimbridge et al., 1992; Andressen et al., 1993), which e.g., in neocortex label subpopulations of interneurons, often in combination with neuropeptides (e.g., Somogyi and Klausberger, 2005).

2 IHC is based on antibodies and allows demonstration of the cellular and ultrastructural localization of peptide/proteins in the microscope. The method was introduced already in the early 1940s by Coons et al. (1942) but was not applied to the nervous system until almost 30 years later (Geffen et al., 1969). Since peptides are rapidly transported out from the cell body after synthesis, the mitosis inhibitor and axonal transport-blocker colchicine is often needed to visualize cell bodies in the brain with this method (Barry et al., 1973; Ljungdahl et al., 1978). Using RIA, also based on (actually often the same) antibodies, developed by Yalow and Berson (1959) almost 60 years ago, concentrations/levels of peptides/proteins can be quantified in tissues and fluids. ISH, also a histochemical technique, detects nucleic acid sequences in tissue sections (Brahic and Haase, 1978; Gee et al., 1983). Since transcripts (mRNAs) are detected, the signal labels cell soma (and to some extent dendrites). The PCR method was invented by Mullis et al. (1987). A note of concern: In addition to specificity problems, especially associated with IHC and GPCRs, histochemical techniques often lack sensitivity to detect low-abundance molecules. Evidence for this view is provided by single cell analysis (Eberwine and Bartfai, 2011). This is particularly true for receptor transcripts, since these proteins have a low turnover (in any case compared to releasable molecules like neuropeptides). And only few receptor molecules are needed for signaling. The present review may ‘underestimate’ the number of molecules that coexist in a neuron and its signaling.

3 The preparation used in Fried et al. (1985) was very suitable for the purpose: The muscle layer of rat vas deferens contains a dense network of noradrenergic nerve terminals storing NPY (Figures 2A–C). However, the very thick, compact smooth muscle layer makes isolation of nerves/storage vesicles difficult. This obstacle was circumvented by castrating rats which leads to muscle atrophy. Thus, fairly pure fractions containing, respectively, synaptic and large vesicles, many of the latter with a visible dense core (Figure 2E), could be obtained (Fried et al., 1985).

4 Of note, Figure 24–4A in Chapter 24 by A. I. Basbaum and T. M. Jessell shows an electron micrograph of an afferent C fiber nerve ending making a type 1 synapse with a dendrite in the *monkey* superficial dorsal horn. Here a string of LDCVs are seen close to the presynaptic membrane opposite to the postsynaptic density. It is not possible to definitely decide, if the LDCVs reach the presynaptic membrane. Nevertheless, the ‘rule’ of extrasynaptic release of LDCVs may not be without exceptions. The micrograph is by courtesy of H. J. Ralston, III. [from the Fourth Edition of the Textbook “Principles of Neural Science” (2000), edited by E. R Kandel, J. H. Schwartz and T. M. Jessell.]

5 First to be identified was the galanin message-associated peptide (GMAP), a product generated from the same precursor as galanin (Rokaeus and Brownstein, 1986). In brain it was also recognized that the N-terminal fragment galanin (1–16), conserved throughout species, is recognized by high affinity receptor sites in the forebrain (Fisone et al., 1989), and subsequently other fragments have been identified in the brain (Sillard et al., 1992; Ihnatko and Theodorsson, 2017). Almost 20 years ago the galanin-like peptide (GALP) was discovered in the porcine hypothalamus and shown to be an endogenous ligand of GalR2 (Ohtaki et al., 1999). GALP (9–21) is identical to galanin (1–13) with a high sequence homology among species. In the analysis of ganglioneuroma tissues Santic and colleagues discovered a splice variant of GALP mRNA, a 25 amino acid peptide and named it Alarin (Santic et al., 2006). This peptide, however, does not bind to any of the three galanin receptors, but still is considered a member of the galanin family (Lang et al., 2015). More recently it was found that spexin, a 14-amino acid peptide, is a ligand at the GalR2 and -R3 receptors (Kim et al., 2014).

6 Please note that the samples from the five regions contain a heterogeneous collection of neurons (and of course glia), where members of the galanin family are present to a varying percentage. (These are cell bodies, since transcripts are monitored. However, the efficacy of probes may vary, and false negatives are not unlikely.) The “LC” sample contains a fairly high percentage of NA neurons. In “DRN” the 5-HT neurons probably represent a small proportion of all neurons, also since the sample includes the vPAG, and more. In the “MRN” sample an even smaller percentage of the neurons are serotonergic. Knowledge of the localization of transcripts of the galanin family is available only for DRN and LC, and even here only to a limited extent.

7 In the early electrophysiological in vitro experiments on rat LC neurons in slices, a marked and lasting (20 min) desensitization developed after application of galanin (Pieribone et al., 1995). If this is true also for GalR3 in the human LC is not known; nor how such an effect, if present, would contribute to/would be of importance for the results obtained in our study on depressed suicides (Barde et al., 2016), and for the present hypothesis.

8 Monoclonal antibodies were ‘invented’ more than 40 years ago (Kohler and Milstein, 1975; Milstein, 1990), and have during the last decades become indispensable tools for treatment of an array of diseases.
